# Breast cancer chemoprevention pharmacogenomics: Deep sequencing and functional genomics of the *ZNF423* and *CTSO* genes

**DOI:** 10.1038/s41523-017-0036-4

**Published:** 2017-08-21

**Authors:** Duan Liu, Ming-Fen Ho, Daniel J. Schaid, Steven E. Scherer, Krishna Kalari, Mohan Liu, Joanna Biernacka, Vivien Yee, Jared Evans, Erin Carlson, Matthew P. Goetz, Michiaki Kubo, D. Lawrence Wickerham, Liewei Wang, James N. Ingle, Richard M. Weinshilboum

**Affiliations:** 1 Division of Clinical Pharmacology, Department of Molecular Pharmacology and Experimental Therapeutics, Mayo Clinic, Rochester, MN USA; 2 Division of Biomedical Statistics and Informatics, Department of Health Sciences Research, Mayo Clinic, Rochester, MN USA; 3 Human Genome Sequencing Center, Baylor College of Medicine, Houston, TX USA; 4 Department of Biochemistry, Case Western Reserve University, Cleveland, OH USA; 5 Division of Medical Oncology, Mayo Clinic, Rochester, MN USA; 6 RIKEN Center for Integrative Medical Science, Yokohama, Japan; 7 Section of Cancer Genetics and Prevention, Allegheny General Hospital and the National Surgical Adjuvant Breast and Bowel Project (NSABP), Pittsburgh, PA USA

## Abstract

Our previous GWAS using samples from the NSABP P-1 and P-2 selective estrogen receptor modulator (SERM) breast cancer prevention trials identified SNPs in *ZNF423* and near *CTSO* that were associated with breast cancer risk during SERM chemoprevention. We have now performed Next Generation DNA sequencing to identify additional SNPs that might contribute to breast cancer risk and to extend our observation that SNPs located hundreds of bp from estrogen response elements (EREs) can alter estrogen receptor alpha (ERα) binding in a SERM-dependent fashion. Our study utilized a nested case-control cohort selected from patients enrolled in the original GWAS, with 199 cases who developed breast cancer during SERM therapy and 201 matched controls who did not. We resequenced approximately 500 kb across both *ZNF423* and *CTSO*, followed by functional genomic studies. We identified 4079 SNPs across *ZNF423* and 3876 across *CTSO*, with 9 SNPs in *ZNF423* and 12 in *CTSO* with *p* < 1E-02 that were within 500 bp of an ERE motif. The rs746157 (*p* = 8.44E-04) and rs12918288 SNPs (*p* = 3.43E-03) in intron 5 of *ZNF423*, were in linkage equilibrium and were associated with alterations in ER-binding to an ERE motif distant from these SNPs. We also studied all nonsynonymous SNPs in both genes and observed that one nsSNP in *ZNF423* displayed decreased protein expression. In conclusion, we identified additional functional SNPs in *ZNF423* that were associated with SNP and SERM-dependent alternations in ER binding and transcriptional regulation for an ERE at a distance from the SNPs, thus providing novel insight into mechanisms of SERM effect.

## Pharmacogenetics: Sequencing finds DNA variants affecting chemoprevention response

DNA sequencing has revealed genetic variants that explain inherited variation in responses to preventative drug therapy for breast cancer. Richard Weinshilboum from the Mayo Clinic in Rochester, Minnesota, USA, and colleagues previously showed that genetic variations in or near two genes—*ZNF423* and *CTSO*—affected how well the drugs tamoxifen and raloxifene reduced the rate of breast cancer occurrence. Building on that finding, Weinshilboum’s team has now sequenced these two genes in 199 patients who developed breast cancer during chemopreventative drug therapy and 201 patients who did not. They identified around 4000 single-nucleotide polymorphisms across each gene, 21 of which were close to estrogen response element (ERE) motifs to which ER﻿α﻿ binds. Functional studies pointed to molecular ways in which some of these gene variants alter drug activity.

## Introduction

Breast cancer is the most common invasive cancer of women worldwide. Treatment of the approximately 70% of women with estrogen-receptor (ER) positive breast cancer with the selective estrogen receptor modulator (SERM) tamoxifen can reduce disease recurrence by nearly 50%.^1^ SERMs, including tamoxifen and raloxifene, have also been shown to be effective agents for the prevention of breast cancer, reducing its occurrence in women at high risk for developing breast cancer by approximately 38%.^2^ The largest and most influential of the SERM breast cancer prevention trials were the double-blind placebo-controlled National Surgical Adjuvant Breast and Bowel Project (NSABP) P-1 trial of tamoxifen and the double-blind NSABP P-2 trial comparing tamoxifen with raloxifene,^3–5^ studies that involved more than 33,000 women and were the basis for United States Food and Drug Administration (FDA) approval of these two drugs for the prevention of breast cancer in high risk women, as well as recent calls by the US Preventive Services Task Force and the National Institute for Health and Care Excellence (NICE) in the UK for the treatment of appropriate high risk women with these drugs in the prevention setting.

**Fig. 1:**
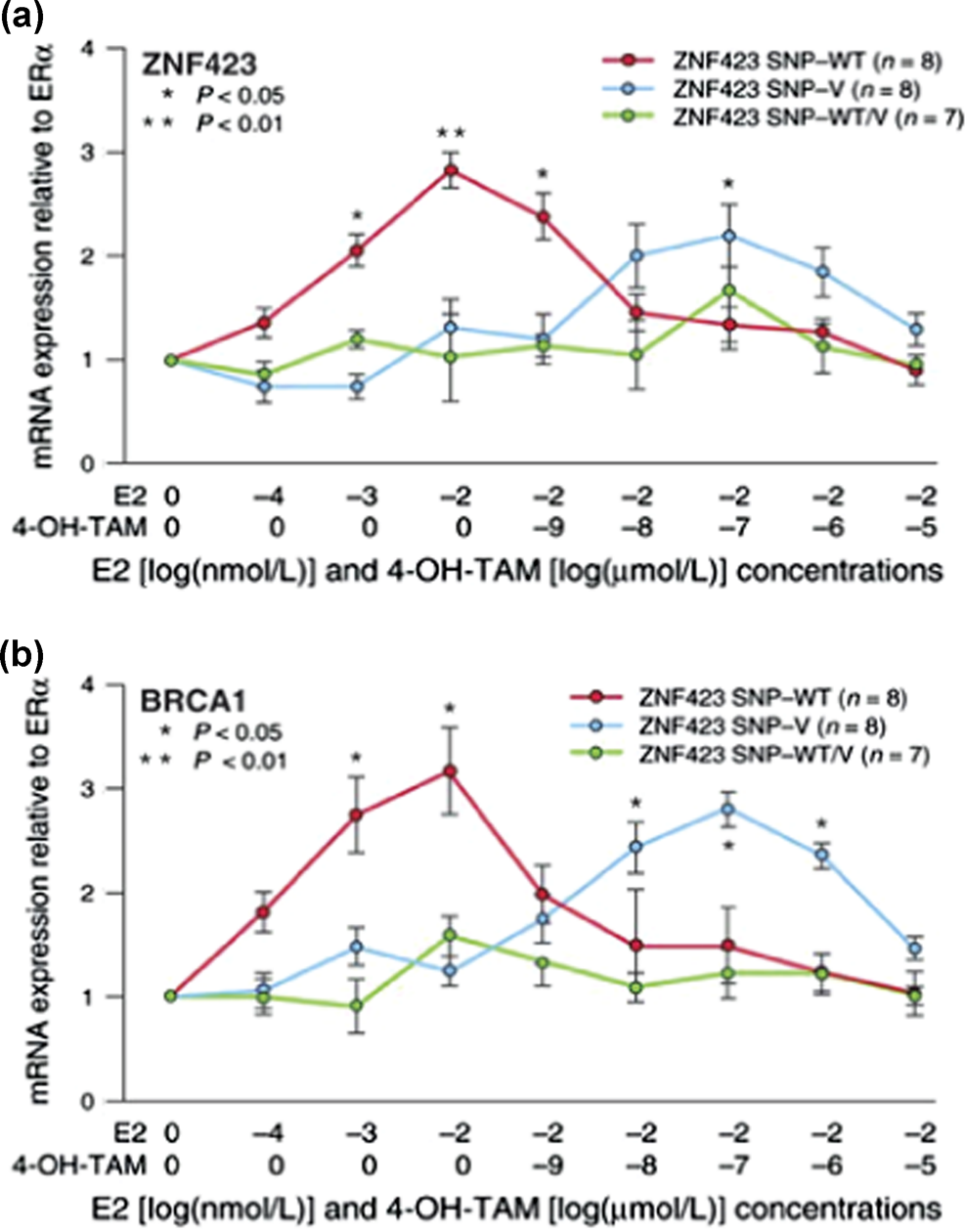
The SERM-dependent, SNP-dependent “reversal” of estradiol induction of *ZNF423* and BRCA1 expression in LCLs. **a** Estradiol (E2) and 4-hydroxytamoxifen (4OH-TAM) dose response curves for expression of ZNF423 (**a**) and BRCA1 (**b**) in LCLs with known genotypes for the *ZNF423* intron 2 rs9940645 SNP. Values are mean ± SEM for 8 determinations. (modified from Fig. 3, Ingle et al. Cancer Discovery 2013^6^)

We recently performed a discovery genome-wide association study (GWAS) using DNA from women who received tamoxifen or raloxifene during the P-1 and P-2 breast cancer prevention trials with a phenotype of breast cancer occurrence during 5 years of SERM preventive therapy.^6^ That GWAS identified two top single nucleotide polymorphism (SNP) signals that mapped to the second intron of the zinc finger protein 423 *(ZNF423)* gene on chromosome 16 and 5’ of the cathepsin O (*CTSO)* gene on chromosome 4. Functional genomic studies demonstrated SNP-dependent estradiol (E2) induction of the expression of both *ZNF423* and *CTSO* and, downstream, of BRCA1, offering a mechanistic explanation related to the role of BRCA1 in DNA double strand break repair for the association of these SNPs with risk for breast cancer during SERM therapy.^6^ Alteration in the regulation of BRCA1 might be expected to influence carcinogenesis for ER-positive tumors as well as ER-negative breast cancers. In addition, and surprisingly, the SNPs in *ZNF423* displayed SERM and SNP-dependent “reversal” of the E2 induction of both *ZNF423* and *BRCA1*—with induction of expression in subjects with variant SNP genotypes during SERM exposure that was associated with decreased breast cancer risk, and the opposite effect in subjects with a “wild type” (WT) SNP genotype for rs9940645, a SNP that mapped 200 bp distant from an estrogen response element ﻿(ERE). This same pattern, was reflected by an identical SNP-dependent difference in expression for BRCA1 (see Fig. 1). E2 also induced the expression of CTSO, and WT genotypes for SNPs near the *CTSO* gene with low *P* values were associated with the E2-dependent induction of CTSO expression and, subsequently, BRCA1 in the presence of estrogen, but this induction did not occur in the presence of variant *CTSO* SNP genotypes, probably because one of the SNPs (rs6813983) near that gene disrupted an ERE.^6^ Variant sequences for the *CTSO*-related SNPs were associated with increased risk for breast cancer. These results indicated that the two top SNP signals observed during the P-1 and P-2 GWAS were linked to the effects of SERM therapy and that they were biologically plausible as a result of their effect on the expression of a major breast cancer risk gene, *BRCA1*.^6^

This series of observations raised the possibility of more highly individualized SERM breast cancer chemoprevention. However, even though SERMs have been approved by the US FDA for the prevention of breast cancer in high risk women, they are not widely used for that purpose because approximately 50 women must be treated to prevent one case of breast cancer and because of rare but serious side effects.^7^ In the present study, we performed Next Generation Sequencing (NGS) across approximately 500 kb of DNA sequence surrounding the SNP signals for both *ZNF423* and *CTSO* using DNA samples from our GWAS study to screen for additional variants associated with breast cancer risk during SERM prevention therapy and also to study the functional implications of those variants—in particular to determine whether the striking SERM-related “reversal” of SNP-dependent variation in the expression of ZNF423 and BRCA1 shown in Fig. 1 for a SNP at a distance from an ERE might be observed for SNPs near—but not within—EREs other than those that we had originally observed in intron 2 of *ZNF423*.

## Results

The purpose of these experiments was to use Next Generation Sequencing (NGS) to characterize genomic sequence variation in areas surrounding the two “top” SNP signals identified during a GWAS performed with DNA samples from the NSABP P-1 and P-2 SERM breast cancer chemoprevention trials [6]. Specifically, we identified and functionally characterized additional genomic sequence variants that had not been observed during GWAS genotyping by deep sequencing approximately 500 kb across both the *ZNF423* gene on chromosome 16 and the *CTSO* gene on chromosome 4. An additional goal was to determine whether the functional effect that we had observed for a SNP, rs9940645, at a distance from an ERE motif in intron 2 of *ZNF423* might be generalized beyond that single SNP. Since the clinical effect of the SERMs used to treat the subjects enrolled in the P-1 and P-2 trials depended on their binding to ERα and the subsequent binding of ERα to EREs, we began by identifying putative EREs in the areas resequenced across both genes to determine whether any SNPs that we observed during resequencing might either create or disrupt an ERE—as we had observed for the rs6810983 SNP near *CTSO*.^6^ In addition, the rs9940645 SNP in *ZNF423* that we observed to be associated with SERM-dependent “reversal” of the expression of ZNF423 in our original GWAS was located approximately 200 bp away from an ERE, so we also used ChIP assays to determine whether any of the SNPs that we observed during resequencing that were near (i.e., ± 500 bp) ERE motifs might alter the functional effect of SERMs in the same way that *ZNF423* rs9940645 did, i.e., whether they might result in an alteration in ER binding in the presence of a SERM. We also functionally characterized all non-synonymous SNPs (nsSNPs) observed during the resequencing of both *ZNF423* and *CTSO*. If these nsSNPs influenced protein quantity or mRNA expression they might also have functional implications.

We found that the resequencing supported the results reported for our original GWAS, but that two SNPs in intron 5 of *ZNF423*, SNPs that were hundreds of bp away from an ERE motif, displayed behavior similar to that of the intron 2 rs9940645 SNP that we observed during the initial GWAS,^6^ raising the possibility that this type of effect for SNPs at a distance from EREs might be relatively common. In addition, during resequencing we identified 10 nsSNPs in *ZNF423* and one in *CTSO* (Supplementary Table 1). Since nsSNPs can alter the expression, function, and/or stability of the encoded protein, we performed site-directed mutagenesis for all nsSNPs observed in both genes to create expression constructs that we used to determine whether the expression of those variant allozymes might be altered. The results of all of these studies are described in subsequent paragraphs.

### DNA sequence variation across *CTSO* and *ZNF423*

Clinical characteristics of the 400 P-1 and P-2 participants for whom we resequenced DNA are listed in Supplementary Table 2. The target region resequenced on chromosome 4 covered 499,868 bp with 4488 SNP positions, and the target region resequenced on chromosome 16 was 550,164 bp in length, with 4553 SNP positions. For both target regions, the depth of coverage was high, with a median depth, over all subjects and all positions, of 291X and 299X for the chromosome 4 and 16 resequenced regions, respectively. Nucleotide variants were filtered based on whether they were monomorphic, missing in more than 10% of participants, or represented genotype calls that were discordant between the Baylor and Mayo TREAT workflows (see Supplementary Figure 1). By requiring 100% agreement between the Baylor and the Mayo TREAT workflow genotype calls, we retained the highest quality variants for analyses, even though many of the discordant variants had concordance rates in the 80–99% range. As a result, the final number of analyzed variants was 3876 for chromosome 4 and 4079 for chromosome 16. The fraction of very rare variants (MAF ≤ 0.005) was 50% for chromosome 4 and 60% for chromosome 16. The percentages of less common variants (MAF 0.005–0.01) were 3.4% for chromosome 4 and 4.8% for chromosome 16, and the percentages of variants with MAF > 0.01 were 46.6% for the chromosome 4 region and 35.1% for chromosome 16.

Results obtained by comparing the frequencies of variants between cases and controls for the chromosome 4 target region are shown graphically in Supplementary Figure 2. The weighted analysis for chromosome 4 shown in Supplementary Figure 2A emphasizes newly discovered variants that were correlated with the initial GWAS top genotyped SNP, rs6835859, depicted in Supplementary Figure 2A by the “G” (result for rs6835859), and the association signals directly beneath it. In contrast, the unweighted analysis in Supplementary Figure 2B shows associations for newly discovered chromosome 4 variants after adjusting for the effect of the rs6835859 SNP. Similar association results for the chromosome 16 target region across *ZNF423* are shown in Supplementary Figure 3. The unweighted analyses replicated the original GWAS association signal (“G” in Supplementary Figure 3A represents the top GWAS genotyped SNP, rs8060157), with a few additional variants detected by sequencing that were in strong linkage disequilibrium (LD) with rs8060157. We should point out that the unweighted analyses in Supplementary Figure 3B show association signals in intron 5 at the 3’-end of the *ZNF423* gene at approximately 49.6MB. We will return to two of those SNPs, rs746157 and rs12918288, later because of striking findings for these two SNPs during functional genomic studies of the relationship of SNPs near, but not within EREs to function. Subsequent paragraphs will describe the results of functional genomic studies of SNPs in or near ERE motifs as well as nsSNPs.

### ERE motif analyses

We performed a series of experiments to pursue and extend our observation that the rs6810983 SNP on chromosome 4 had a functional effect on *CTSO* transcription by disrupting an ERE sequence motif, while the variant genotype for the *ZNF423* rs9940645 SNP on chromosome 16 located approximately 200 bp from an ERE motif in intron 2 of that gene displayed “reversal” of the functional effect of that ERE in the presence of a SERM. Specifically, we set out to determine whether this type of SNP-dependent response might be a general phenomenon—i.e., differential SNP effects on ERα binding to EREs at a distance from the SNP, with SERM-induced “reversal” of both ER binding and gene expression.

As a first step, we attempted to identify all possible ERE sequence motifs in the areas resequenced across *ZNF423* and *CTSO*. The method used to identify “putative” EREs, as described in the Methods, identified 208 possible ERE motifs across the area resequenced for *ZNF423* and 118 across the area resequenced for *CTSO*. In addition, 1294 SNPs were located within ± 500 bp of putative EREs across the *ZNF423* gene, and 815 SNPs were within ± 500 bp of putative EREs in the area sequenced across or near the *CTSO* gene. SNPs predicted to either create or disrupt ERE motifs in either of the areas resequenced are listed in Supplementary Tables 3 and 4, but none of the SNPs listed in those Tables displayed associations with the occurrence of breast cancer during SERM therapy with *p* values ≤ 1E-02. However, when we performed a similar exercise for the association of SNPs located within ± 500 bp of putative ERE motifs with breast cancer occurrence during 5 years of SERM therapy, 9 SNPs near but not within 7 putative EREs across the *ZNF423* gene (Table 1) and 12 SNPs near but not within 6 putative EREs in the region surrounding the *CTSO* gene were associated with breast cancer occurrence with *p* < 1E-02 (Table 1). We next performed experiments designed to determine whether these putative ERE motifs were functional (i.e., whether they could bind ERα in the presence of E2) and, if so, whether any of them might display SNP-dependent SERM-dependent alteration in ERα binding similar to that which we had observed for rs9940645 in *ZNF423* intron 2.^6^

**Table 1:** SNPs across the *ZNF423* gene on chromosome 16 and SNPs across the *CTSO* gene on chromosome 4 within ±500 bp of ERE motifs with *p* values ≤ 10E-03. *p* values are for associations with the occurrence of breast cancer during SERM therapy

SNP rs ID	SNP Position on Chr.16	Motif Sequence	Motif Start Position	Motif End Position	Strand	Distance from Motif	MAF (400)	MAF (all samples)	Common.Variant	*p* Value
rs7187662	49614038	GTTGGTCTGGATGACTC	49613770	49613787	−	250	0.20125	0.219389	T	6.11E-04
rs72780324	49691162	CATGGATTCCCTGACCT	49690861	49690878	+	283	0.05250	0.049403	A	7.03E-04
rs746157	49593393	GAAGGGGCAGCTGACTC	49592974	49592991	−	401	0.17250	0.190402	T	8.44E-04
rs12925456	49596645	GATGGTCTTGATCTCCT	49596906	49596923	+	261	0.17125	0.189488	T	1.17E-03
rs11642983	49597423	GATGGTCTTGATCTCCT	49596906	49596923	+	499	0.17125	0.189488	T	1.17E-03
rs72780328	49708387	CAGAGCCACCCTGTCCT	49708366	49708383	−	3	0.03625	0.035656	C	1.98E-03
rs71382759	49600680	GATGAGCTAGCTCACCC	49601144	49601161	+	464	0.09375	0.115006	C	3.04E-03
rs12918288	49593188	GAAGGGGCAGCTGACTC	49592974	49592991	−	196	0.09625	0.116332	G	3.43E-03
rs57148286	49688324	CAGGGACAGCATGAGCT	49688371	49688388	−	47	0.05875	0.063394	G	6.23E-03

SNP rs ID	SNP Position on Chr.4	Motif Sequence	Motif Start Position	Motif End Position	Strand	Distance from Motif	MAF (400)	MAF (all samples)	Common.Variant	*p* Value
rs1490555	157251916	CTGTTTCAGCTTGACTT	157251780	157251797	+	118	0.18125	0.175165	T	3.83E-03
rs1490556	157251977	CTGTTTCAGCTTGACTT	157251780	157251797	+	179	0.18125	0.175165	A	3.83E-03
rs1873358	157252132	CTGTTTCAGCTTGACTT	157251780	157251797	+	334	0.18125	0.175165	A	3.83E-03
rs2879978	157252282	CTGTTTCAGCTTGACTT	157251780	157251797	+	484	0.18125	0.175165	G	3.83E-03
rs10010729	157248748	GAAGGGTAAGAGGAACT	157248919	157248936	−	171	0.16875	0.165087	G	5.09E-03
rs1490554	157251896	CTGTTTCAGCTTGACTT	157251780	157251797	+	98	0.16875	0.165087	A	5.09E-03
rs2101586	157261188	CTGGGTTTACATGACCT	157261391	157261408	+	203	0.16875	0.165087	C	5.09E-03
rs6536168	157261438	CTGGGTTTACATGACCT	157261391	157261408	+	29	0.16875	0.165087	C	5.09E-03
rs4691210	156919006	TTAGGGTAGAATGACTC	156918505	156918522	+	483	0.02125	0.034025	T	7.99E-03
rs4691214	156923110	CAAAGCCAGCTGGACTT	156923087	156923104	+	5	0.02125	0.034025	A	7.99E-03
rs4691216	156923435	CATGGTCTCGCTGACTT	156923576	156923593	−	141	0.02125	0.034025	C	7.99E-03
rs4234895	156923944	CATGGTCTCGCTGACTT	156923576	156923593	−	350	0.02125	0.034025	T	7.99E-03

### ChIP assays for ERα binding to ERE motifs

We next studied the association of SNP genotypes with ERα binding to ERE motifs within ± 500 bp of the SNP after treatment with vehicle, E2, 4OH-TAM or E2 plus 4OH-TAM for SNPs by performing ChIP assays. We limited the SNPs studied to those within +/− 500 bp from ERE motifs because we found that SNPs more than 500 bp from EREs could not be detected reliably when using ChIP DNA as a template because of the sonication and immunoprecipitation steps and the fact that large PCR amplicons failed to reach optimal amplification efficiency. Nine SNPs near 7 putative EREs across the *ZNF423* gene (Table 1) and 12 SNPs near 6 putative EREs in the region surrounding the *CTSO* gene (Table 1) were studied in this fashion. Since we had found that SNPs that were more than 500 bp distant from the ERE could not be detectable reliably by qPCR when using ChIP DNA as a template, we did not include the rs11642983 and rs71382759 SNPs in *ZNF423*, or rs28799978, rs4691210 and rs4234895 SNPs in *CTSO* for the ChIP-qPCR studies. The concentrations of E2 and 4OH-TAM used to perform these experiments were optimal based on dose-response curves performed during functional genomic studies of the top SNPs for our original GWAS.^6^ Combination treatment with E2 plus 4OH-TAM was used to simulate the clinical situation in which both estrogen and SERM would be present.

**Fig. 2:**
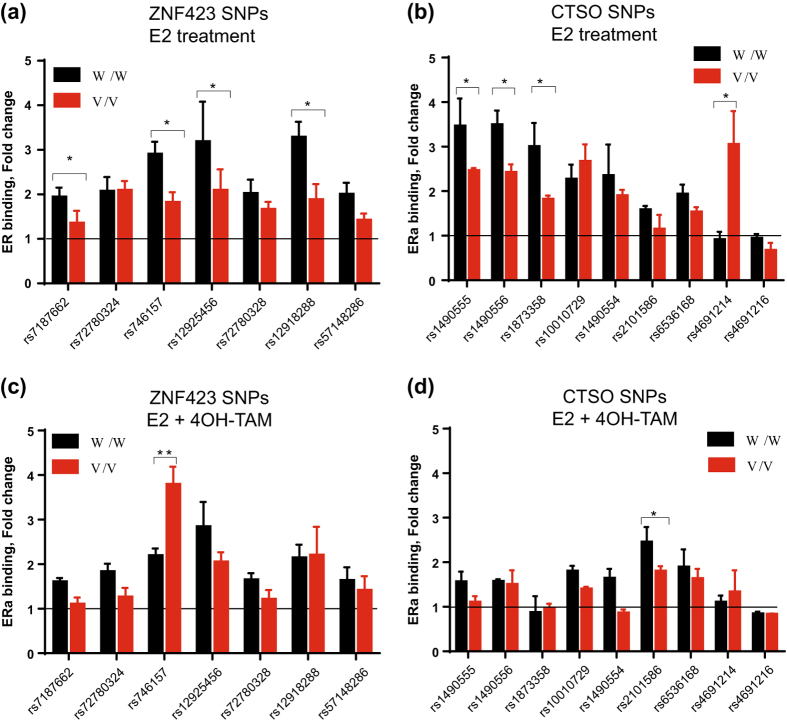
ChIP assays showing fold changes in ERα binding to DNA sequences containing *ZNF423* SNPs (panels **a** and **c**) or *CTSO* SNPs (panel **b** and **d**). ChIP assays were performed in LCLs homozygous for WT (W/W) or variant (V/V) genotypes for these SNPs after exposure to E2 (0.01 nM) or E2 (0.01 nM) plus 4OH-TAM (0.01 μM). Percentage of ChIP DNA/input was determined by qPCR. The level of enrichment was expressed as relative enrichment above vehicle treatment. The values shown represent mean ± SEM for six determinations.*P < 0.05, **P < 0.005 comparing WT and V SNP genotype cell lines at the same concentrations of E2 and 4OH-TAM

**Fig. 3:**
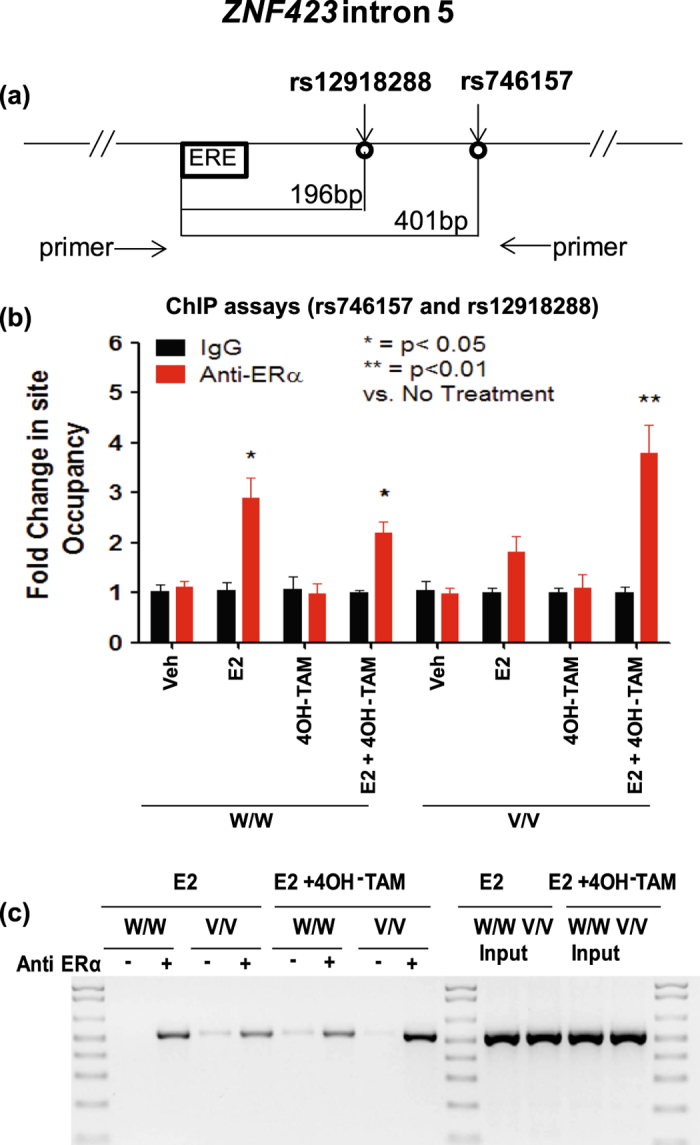
ChIP assay results for the rs746157 and rs12918288 SNPs in *ZNF423* intron 5. **a** The figure shows a schematic representation of the ERE motif located near the rs12918288 and rs746157 SNPs in intron 5 of *ZNF423*. **b** Bar graphs showing quantitative ERα ChIP results for the area of ZNF423 containing rs746157 and rs12918288. The values shown represent mean ± SEM for six determinations. **c** Agarose gel assay showing differential binding for the region including the rs746157 and rs12918288 SNPs for LCLs homozygous for WT (W/W) or variant (V/V) genotypes for these two SNPs after exposure to E2 (0.01 nM) or E2 (0.01 nM) plus 4OH-TAM (0.01 μM)

We observed that, in the presence of E2 alone, 4 SNPs near 4 ERE motifs in the *ZNF423* gene (Fig. 2a), and 4 SNPs near 2 ERE motifs close to the *CTSO* gene (Fig. 2b) displayed significant SNP-dependent ERα binding (*P* < 0.05) even though none of these SNPs was within an ERE motif. In order to determine whether the putative ERE sequence motifs that we studied in the lymphoblastoid cell lines (LCLs) would also bind to ER in breast cancer cell lines in the presence of E2, we repeated the ChIP assays in the presence of vehicle and E2 using two ER + breast cancer cell lines—ZR-75-1 and MCF-7. As shown in Supplementary Figure 6, 6 out of 7 of the ERE motifs showed E2 dependent binding for the *ZNF423* sequences in both cell lines, and for the *CTSO* sequences it was 6 of 9 in one cell line and 5 of 9 in the other. For the LCLs, in addition to E2 exposure, we also studied the cells after treatment with E2 plus 4OH-TAM. In those experiments, 5 of 6 functional *ZNF423* and all of the functional *CTSO* EREs either lost ERα binding or the binding displayed a striking decrease, as anticipated (Fig. 2c and d). However, the ERE near rs746157 in intron 5 of *ZNF423* displayed a striking reversal of the binding pattern in the presence of 4OH-TAM, showing increased binding in the presence of the variant rather than the WT SNP genotype (Fig. 2c)—similar to what we had observed for rs9940645 in our original GWAS. The rs746157 SNP (*p* = 8.44E-04 for association with breast cancer risk) mapped 401 bp from a functional ERE in intron 5 of the *ZNF423* gene, as shown graphically in Fig. 3a. The ERE near this SNP displayed a striking increase in ERα binding after treatment with E2 plus 4OH-TAM (*P* < 0.01 when compared with vehicle treatment) (Fig. 3b and c). It should be noted that the PCR amplicon for rs746157 also included the rs12918288 SNP because these two SNPs are located close together in intron 5 of *ZNF423* and they are in tight LD (*r* = 0.99*)*. This pair of SNPs is located 238,000 bp distant from the rs9940645 SNP in intron 2 of *ZNF423* that we had originally shown to be associated with enhanced ERα binding for the variant allele after treatment with E2 plus 4OH-TAM in our original P-1 and P-2 GWAS.^6^ In addition, the two *ZNF423* intron 5 SNPs (rs746157 and rs12918288) were not in LD with the intron 2 rs9940645 SNP, suggesting that any functional effects of the intron 5 SNPs would be independent of functional effects mediated by the rs9940645 SNP in intron 2. We next tested the possible effect of the rs746157 and rs12918288 SNPs on the expression of ZNF423 and, downstream, the expression of BRCA1.

### Intron 5 SNPs and SERM-dependent expression of ZNF423 and BRCA1

**Fig. 4:**
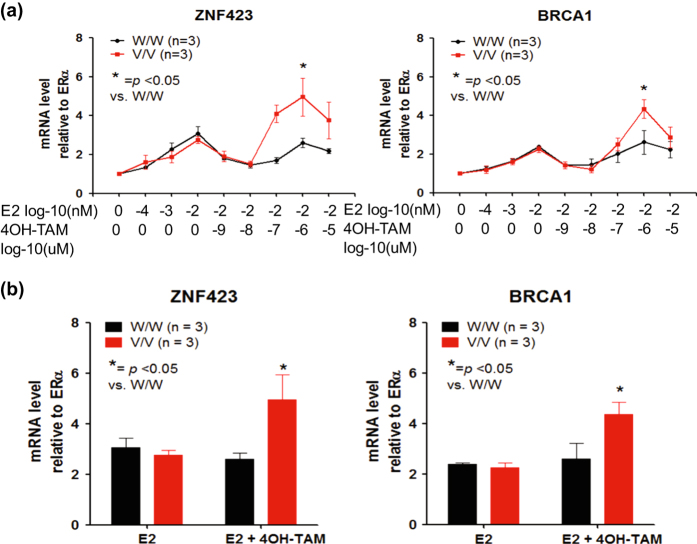
The induction of ZNF423 and BRCA1 mRNA expression in a SNP-dependent, SERM-dependent fashion for LCLs homozygous for WT and variant *ZNF423* rs746157 and rs12918288 genotypes. **a** The figure shows the effect of E2 and E2 plus 4OH-TAM dose response curves on the expression for ZNF423 and BRCA1 in LCLs homozygous for WT and variant genotypes for the rs746157 and rs12918280 SNPs in *ZNF423* intron 5. Values are mean ± SEM for 3 determinations. All of these LCLs were homozygous WT for the intron 2 rs9940645 SNP. **b** Bar graphs showing mRNA expression for ZNF423 and BRCA1 at the optimal concentrations for E2 (0.01 nM) and E2 (0.01 nM) plus 4OH-TAM (0.01 μM) shown in (**a**). Compare with Fig. 1b. Data represented as mean ± SEM

In our original GWAS, enhanced ERα binding to the intron 2 ERE motifs located 200 bp from the rs9940645 SNP was associated with a functional phenotype, increased expression of ZNF423 and, downstream, the expression of BRCA1. We observed that the two SNPs (rs12918288 and rs746157) near the ERE motif in intron 5 of *ZNF423*, as shown graphically in Fig. 3a, exhibited binding behavior similar to that of the ERE 200 bp away from rs9940645 in intron 2. Therefore, we next asked whether these two SNPs in intron 5 of *ZNF423* might also be capable of regulating ZNF423 transcription. The results are shown in Fig. 4. The upper panel shows the effect of increasing concentrations of E2, followed by increasing concentrations of 4OH-TAM on both ZNF423 and BRCA1 mRNA expression in LCLs with WT and variant genotypes for the intron 5 SNPs. It should be emphasized that these LCLs were all homozygous WT for the rs9940645 SNP in *ZNF423* intron 2 to make it possible to isolate the effect of rs746157 and rs12918288 SNPs in intron 5 separate from the effect of the intron 2 SNP. These results can be compared with those shown in Fig. 1a and b for differing genotypes for the rs9940645 SNP in *ZNF423* intron 2. In the case of the intron 5 SNPs, there was not a SNP-dependent difference in the E2-dependent induction of *ZNF423* by E2, but there was significantly greater induction of ZNF423 mRNA expression in LCLs homozygous for variant compared with those homozygous for WT SNP genotypes when 4OH-TAM was present in addition to E2 (Fig. 4a). Furthermore, this pattern was reflected downstream by the induction of BRCA1 mRNA expression, as shown in Fig. 4a and by the bar graphs in Fig. 4b, which depict the level of induction at the optimal concentrations of E2 or E2 plus 4OH-TAM. The gene expression patterns shown in Fig. 4 are compatible with the ChIP assay results for these same SNPs that are shown in Fig. 3b. Therefore, for *ZNF423* intron 5, just as in intron 2, alterations in nucleotide sequences located hundreds of bp from an ERE were associated with striking SNP and SERM-dependent differences in both ERα binding and in the functional effect on ZNF423 expression.

### Functional genomic studies of *ZNF423* and *CTSO* nsSNPs

**Fig. 5:**
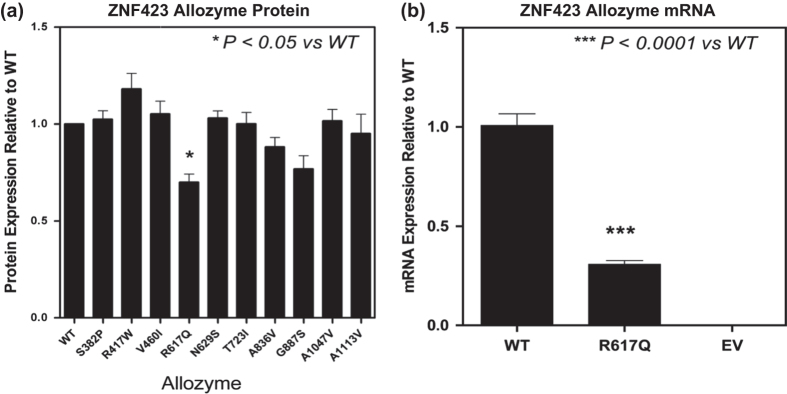
**a** Levels of protein expression in COS-1 cells for *ZNF423* WT and variant allozymes. **b** Levels of mRNA expression in COS-1 cells for the Q617 ZNF423 variant allozyme compared with that for the WT allozyme. Data represented as mean ± SEM for 6 determinations. EV = empty vector

We also identified 10 nsSNPs in *ZNF423* and 1 in *CTSO* which would encode variant allozymes and, as a result, could potentially have functional implications, both for the protein expression of these two genes and for their downstream effect on BRCA1 expression. Therefore, we determined whether the nsSNPs might alter protein quantity, a common effect of nsSNPs.^8^ To do that, we performed site-directed mutagenesis using COS-1 cells and protein expression was quantified by Western blot analysis (Supplementary Figure 4). We observed that one of the *ZNF423* nsSNPs, a SNP that encoded an Arg617Gln (R617Q) substitution, resulted in a significant decrease in ZNF423 protein expression (70 ± 4% of WT, n = 6, *p* < 0.05) (Fig. 5a). The single nsSNP in *CTSO* which resulted in a Ser208Phe (S208F) change in the encoded amino acid sequence failed to alter CTSO protein expression (data not shown).

Accelerated protein degradation is a common mechanism responsible for the decreased level of protein displayed by variant allozyme, either as a result of the effect of ubiquitin-proteasome or autophagy-mediated degradation.^8–11^ To test that possibility for the R617Q ZNF423 allozyme, MG132, a proteasome inhibitor, and the autophagy inhibitor 3MA were incubated with transfected COS-1 cells to determine whether they might alter expressed protein level for the ZNF423 Q617 variant allozyme. However, we failed to observe significant alteration in protein expression as a result of the inhibition of protein degradation by either MG132 or 3MA (Supplementary Figure 5). We then determined whether the decrease in ZNF423 Q617 variant allozyme protein expression might result from a decrease in the level of mRNA using the same cell lines from which the data shown in Fig. 5a were obtained. We observed that the level of mRNA encoding the Q617 variant allozyme was significantly decreased to 30.8 ± 2.0% of that for the WT (n = 6, *p* < 0.0001) (Fig. 5b), suggesting that the decrease in protein expression for the ZNF423 Q617 variant allozyme might be due to decreased mRNA expression rather than proteasome or autophagy-mediated degradation of the encoded protein.

## Discussion

Tamoxifen or raloxifene can reduce the occurrence of breast cancer in women at increased risk for this disease by approximately 50%,^3, 4^ and both drugs have been approved by the FDA for the prevention of breast cancer in these women—primarily on the basis of the results of the NSABP P-1 and P-2 clinical trials.^3, 4^ As a result, the US Preventive Services Task Force and NICE in the UK have recommended that all women at high risk for breast cancer consider preventive therapy with one of these drugs. However, neither of these SERMs is widely prescribed to prevent breast cancer, in part because approximately 50 women must be treated for each case of breast cancer prevented and because of rare,^12^ but potentially serious side effects of SERM therapy.^13^ In an effort to determine whether it might be possible to more highly “individualize” SERM breast cancer prevention, we recently performed a GWAS using DNA samples from women who participated in the P-1 and P-2 clinical trials. That study identified SNP signals in or near the *ZNF423* and *CTSO* genes. We also showed that both genes regulated the expression of BRCA1 in a SNP and SERM-dependent fashion. In the case of *ZNF423*, that process involved a novel mechanism during which SERMs, in the presence of a variant SNP genotype, “reversed” the E2 dependence of ZNF423 and BRCA1 induction, i.e., mRNA expression from both ZNF423 and BRCA1 was induced by E2 when the WT, but not the variant SNP sequence was present, but the reverse was true in the presence of a SERM (Fig. 1). That series of observations raised both mechanistic and clinical questions; specifically, whether there might be other variant DNA sequences in or near *ZNF423* and *CTSO* that might also influence response to SERM chemoprevention therapy and a more general mechanistic question of whether the SERM-dependent “reversal” of the effect of the *ZNF423* intron 2 rs9940645 SNP located approximately 200 bp away from an ERE was unique, or whether there might be other SNPs that behaved in a similar fashion. It should be emphasized that it has been known for some time that DNA sequence variation very near, but not in an ERE motif can alter ERα binding.^14^ However, those studies did not test sequence variation hundreds of bp away from an ERE motif. The results of our GWAS, when combined with the results reported here, raise the question of exactly how SNPs located hundreds of bp distant from ERE motifs are “sensed” and how their effects are mediated. In addition, previous studies of the effect on binding of DNA sequence variation near, but not within ERE sequences usually did not address the possible effects of drugs. The mechanism responsible for the striking “reversal” of SERM response in the presence of the rs99440645 SNP in *ZNF423* remains unexplained. However, in recent preliminary studies we have obtained evidence that ER coregulatory proteins such as CALML3 might contribute to this phenomenon.^15^ The present results indicate clearly that two SNPs in intron 5 of *ZNF423* display a similar SNP and SERM-dependent difference in ERα binding and subsequent gene transcription. The fact that SNPs in two different introns of one gene, *ZNF423*, display this behavior makes it clear that we need to determine what proportion of the approximately 10,000 ERE motifs in the human genome^16–19^ display this type of drug-dependent SNP-dependent difference in receptor binding; what the underlying mechanism(s) might be; and what the implications of this SNP genotype-dependent, drug-dependent effect might be for variation in clinical SERM effect, i.e., what the implications might be for SERM pharmacogenomics.

The present study also has implications with regard to the use of deep sequencing in pharmacogenomics. Next Generation DNA sequencing provides a comprehensive view of the human genome and offers a wealth of information on novel biology. A major rationale for performing this type of study has involved the possible role of rare variants for variation in the phenotype of interest. The present study raises the possibility that an additional application of deep sequencing for pharmacogenomics might involve the definition of variation within DNA binding sequence motifs, in the present case ERα binding motifs. However, a similar approach might also be applied to any nuclear receptor or other transcription factor that binds to specific DNA sequence motifs. Next Generation DNA sequencing, with adequate coverage, might help make it possible to move beyond a focus on individual SNPs, to include variation in sequences encoding transcription factor binding motifs, as demonstrated in this report for EREs, or for SNPs near or hundreds of bp distant from those binding motifs.

Many of the functional studies described in this series of experiments began with the use of an LCL model system that made it possible to select cell lines for study with virtually any common genotype or combinations of genotypes, something which would not be possible using breast cancer cell lines. However, we also used ZR-75-1 and MCF-7 ER positive breast cancer cell lines to confirm the LCL data shown in Fig. 2 (see Supplementary Figure 6). The LCLs, like any model system, have limitations, so the results reported here will have to be replicated by future studies conducted with additional cell lines and with clinical samples. In addition, we focused our ChIP assays on EREs within ± 500 bp of SNPs. That was done because—as described earlier—in order to ensure maximum accuracy, we have found that these assays are best limited to PCR amplicons that range from 100–500 bp in length. However, the results of the present studies have demonstrated clearly that SNPs at a distance from an ERE can have a striking effect on ER binding and subsequent gene transcription. However, it is possible that SNPs located > 500 bp from EREs might also regulate SNP, estrogen and SERM-dependent ER binding and subsequent transcription. Therefore, future studies will be needed both to investigate mechanisms underlying the SNP-estrogen and SERM-dependent effects that we have observed, mechanisms which might contribute broadly to individual variation in SNP-dependent variation in transcriptional regulation and variation in SERM drug response.^15^

## Conclusions

In the present study, we identified additional SNPs that might contribute to inherited variation in SERM breast cancer prevention therapy by deep sequencing across *CTSO* and *ZNF423* and subsequent functional genomic studies. The present results also verify and extend a novel pharmacogenomic mechanism by which SNPs at a distance from a transcription factor binding motif, EREs in this case, can have striking effects on the functional implications of binding. Of perhaps greatest importance is the possibility that these SNP-associated effects might include either the alteration, or even the reversal, of drug effect. All of these possibilities will have to be pursued in the course of future studies if we are to approach the ultimate goal of more highly individualized breast cancer chemoprevention.

## Methods

### Ethics statement

The protocol for this study was reviewed and approved by the Mayo Clinic Institutional Review Board (reference number: MC0831). All cases and controls received a study-specific identifier (an anonymization code number) that permitted the data derived from the DNA samples to be linked to clinical data from the applicable P-1 or P-2 dataset using an “honest broker agent” who was independent from the study. Confidentiality was maintained for all study participants. This study was conducted in accordance with the appropriate protocols. All women whose DNA samples were used in this study gave their consent for participation in the study and for publication of the study results in a peer-reviewed scientific journal.

### Selection of cases and controls from the initial GWAS

Because the case and control samples used in the present study were obtained from our GWAS, the design of that study will be summarized briefly. Cases were women who experienced invasive breast cancer or ductal carcinoma in situ (DCIS), and controls were women who did not experience those breast events during SERM therapy. A nested matched case-control design was used, with matching on the following factors: 1) trial and treatment arm (P-1 tamoxifen, P-2 tamoxifen, P-2 raloxifene); 2) age at trial entry; 3) 5-year predicted breast cancer risk based on the Gail model (<2.00%, 2.01–3.00, 3.01–5.00, >5.01), 4) history of lobular carcinoma in situ; 5) history of atypical hyperplasia in the breast; and 6) time on study (controls had to be on study at least as long as the time to diagnosis of the breast event for the case). Because 94.2% of the participants treated with tamoxifen or raloxifene were Caucasian, our study was restricted to Caucasian participants to minimize population stratification. Genotypes were determined at the RIKEN Center for Integrative Medical Science with the Illumina Human610-Quad BeadChip. A total of 592,236 SNPs were genotyped for the GWAS and 547,356 SNPs were carried forward for analysis after quality control.

In our original GWAS, the minor allele of the rs6835859 SNP on chromosome 4, and the major allele of the rs8060157 SNP on chromosome 16 were each associated with increased risk for breast cancer. Because of the cost required to resequence all 1763 of the DNA samples included in the GWAS, we selected a total of 400 samples for resequencing, 199 from cases and 201 from controls. To select case and control samples optimally while also using information with regard to genotypes for the chromosome 4 and 16 SNPs observed in our GWA study, we stratified the GWAS sample according to case-control status as well as joint genotypes for the SNPs on chromosome 4 and 16 and randomly sampled participants from those 18 strata (see Supplementary Table 5 for additional detail). This type of outcome-dependent two-phase stratified sampling design has been shown to be more efficient than simple random sampling within case/control groups,^20^ particularly when the GWAS SNPs used to stratify samples are in linkage disequilibrium with novel variants detected by sequencing.^21^ Sample preparation, DNA sequencing, library production, capture sequencing and sequence analysis are described in detail in the Supplementary Methods.

### Identification of ERE motifs overlapping and adjacent to SNPs

We used motif-based sequence analysis tools to identify ERE motifs that were either overlapping with or were less than 500 bp away from a SNP. Specifically, we applied the Find Individual Motif Occurrences (FIMO) tool http://meme.nbcr.net/meme/fimo-intro.html to identify ERE motifs. Sequence Alignment/Map (SAM) tools (http://samtools.sourceforge.net/) were used to extract the 500 bp flanking regions from the human reference genome at those sites and to build FASTA files to submit to the FIMO tool. Together with the FASTA file, the FIMO software was also provided with a canonical ERE motif sequence^22^ to identify putative ERE motifs in which a SNP might create, disrupt or change the function of an ERE. After the FIMO results were obtained, we used BEDTools^23^ to calculate the distance from variant sites to the locations of ERE motifs.

### Chromatin immunoprecipitation (ChIP) assays

The lymphoblastoid cell line (LCL) model system used in these studies has been utilized repeatedly to generate and/or test pharmacogenomic hypotheses arising from clinical GWAS.^24–30^ Its use in the present study made it possible to evaluate associations between *ZNF423* and *CTSO* SNP genotypes, ZNF423 expression and the downstream effect on the expression of BRCA1. Specifically, LCLs with known genotypes for the *ZNF423* or *CTSO* SNPs that were associated with breast cancer occurrence were cultured as described previously.^6^ Before E2 and/or 4-hydroxytamoxifen (4OH-TAM) treatment, the cells were cultured for 24 h in RPMI containing 5% (v/v) charcoal-stripped FBS, followed by 24 h of starvation in FBS-free RPMI media. Cells were harvested after treatment for 24 h with E2 (0.01 nM) and/or 4OH-TAM (10^−6^μM). ChIP assays were performed using the EpiTect® ChIP One-Day Kit (QIAGEN, Germantown, Maryland, USA). The anti-ERα antibody used for immunoprecipitation was obtained from Thermo Scientific (Rockford, Illinois, USA). Each purified ChIP DNA sample (2 μl) was added to PCR reaction mixtures with a final volume of 20 μl. The sequences of primers used to amplify target sequences that contained the SNPs of interest are listed in Supplementary Table 6. After amplification, 10 μl of PCR product was loaded on 2% agarose gels and subjected to electrophoresis at 80 mA in 1 × TAE buffer.

### Drug treatment

Three LCLs homozygous for WT genotypes for the intron 5 SNPs rs746157 and rs12918288 in *ZNF423*, and three cell lines homozygous for the variant genotypes for those two SNPs were used to perform these experiments. All LCLs used in these experiments were homozygous for the WT genotype for the intron 2 rs9940645 SNP to prevent confusion based on possible effects of the *ZNF423* intron 2 SNP. Before E2 treatment, the LCLs were cultured as described above. After serum starvation, 2 × 10^6^ cells from each cell line were cultured for 24 h in 6-well plates in RPMI-1640 media that contained 0, 0.0001, 0.001, and 0.01nmol/L E2. 4OH-TAM was then added to the same media containing 0.01nmol/L E2 with final 4OH-TAM concentrations of 10^−9^, 10^−8^, 10^−7^, 10^−6^, and 10^−5^μmol/L, and the cells were cultured for an additional 24 h. Total RNA was then isolated from the cells using the RNeasy® Plus Mini Kit (Qiagen). mRNA expression was determined by qRT-PCR.

### Site-directed mutagenesis for non-synonymous (ns) SNPs

The wild type (WT) human ZNF423 (NM_015069) and CTSO (NM_001334) cDNAs were obtained from OriGene Technologies, Inc. (Rockville, Maryland, USA) and were subcloned into the eukaryotic expression vector pCMV6-XL4. The inserts were sequenced in both directions to verify their sequences. Site-directed mutagenesis was then performed using the QuikChange II kit (Stratagene, La Jolla, California, USA) to create expression constructs for each of the variant allozymes that had been observed. Sequences of the primers used to perform site-directed mutagenesis are listed in Supplementary Table 1. The sequences of variant constructs were confirmed by DNA sequencing. COS-1 cells were then transfected using the Fugene HD Transfection reagent (Promega, Madison, Wisconsin, USA) at a charge ratio of 3:1 with expression constructs encoding WT and variant ZNF423 and CTSO allozymes, as well as a pCMV6-XL4 empty vector (EV) that served as a negative control. The cells were also co-transfected with pSV-ß-galactosidase (Promega, Madison, Wisconsin, USA) to correct for variation in transfection efficiency. After 48 h, the cells were lysed using the Report Lysis Buffer Kit (Promega, Madison, Wisconsin, USA) and ß-galactosidase activities were assayed using the ß-galactosidase assay system.

### Gene expression quantification

COS-1 cells were transfected with plasmids encoding *ZNF423* WT and R617Q variant allozymes as described below for the protein degradation studies. After 24 h, whole cell lysates from the same cells were prepared for Western blot analysis. Specifically, cell lysate supernatants from the transfected cells were assayed for ß-galactosidase activity, and the samples were subjected to electrophoresis on 7.5% TGX gels. After electrophoresis, proteins were transferred to PVDF membranes (Bio-Rad, Hercules, California, USA), and the membranes were incubated with rabbit polyclonal anti-ZNF423 (Santa Cruz Biotechnology, Santa Cruz, California, USA) or purified mouse anti-CTSO antibody (R&D Systems, Minneapolis, Minnesota, USA) diluted 1:500, followed by incubation with a secondary antibody (1:10,000). Immunoreactive proteins were detected using the ECL Western Blotting System (Thermo Scientific, Rockford, IL) and were quantified using the ChemiDoc™ XRS + System (Bio-Rad, Hercules, California, USA). Data were expressed as percentages of the intensity of WT ZNF423 or CTSO protein on the same gel. ZNF423 mRNA level was quantified by qRT-PCR using the Power SYBR® Green RNA-to-CT™ 1-Step Kit (Life Technologies, Carlsbad, California, USA).

### Protein degradation studies

The *ZNF423* Arg617Gln (R617Q) variant allozyme, an allozyme that displayed a significant decrease in protein quantity when compared to WT, was used to perform protein degradation studies. 24 h after transfection with *ZNF423* WT or Q617 variant constructs, COS-1 cells were transferred from 100mm plates into 6-well plates. To test for possible proteasome-mediated degradation, the cells were then treated for 6–8 h with either DMSO or 20 µM MG132, a proteasome inhibitor, dissolved in DMSO. To determine whether autophagy might participate in degradation of the variant protein, transfected cells were also treated for 48 h with 10 µM 3-methyladenosine (3MA). Immunoreactive ZNF423 protein was quantified by Western blot analysis after correction for transfection efficiency on the basis of β-galactosidase activity.

### Data availability

All data supporting our findings can be found in the main paper or in supplementary files.

## Electronic supplementary material

Supplementary Methods

Supplementary Tables

Supplementary Figures
