# Supplementary material for: Breast cancer chemoprevention pharmacogenomics: Deep sequencing and functional genomics of the ZNF423 and CTSO genes
Source: NPJ Breast Cancer. 2017 Aug 21;3:30. doi: 10.1038/s41523-017-0036-4 (PMC5566425; doi:10.1038/s41523-017-0036-4)
Supplement: Supplementary file 1 — Supplementary Methods [file 41523_2017_36_MOESM1_ESM.docx]

**Supplementary Methods**

**Sample preparation, sequencing library production, and capture sequencing**

DNA was extracted using an Autogen Flexstar with Qiagen Flexigene chemistry. The final DNA concentration of each sample was 0.25µg/µl based on Nanodrop, and normalized aliquots were sent to the Baylor College of Medicine Human Genome Sequencing Center (HGSC) based on picogreen concentrations. Samples were checked at the HGSC for quantity and quality by PicoGreen^®^ assay and by agarose gel electrophoresis, respectively. Illumina paired-end precapture libraries were constructed using a modification of the manufacturer’s protocol.

**Next Generation DNA Sequencing and Sequence Analysis**

Subsequent paragraphs provide detailed descriptions of the Next Generation DNA sequencing performed for this study and of the subsequent analysis of that sequence, both at Baylor and at the Mayo Clinic. Specifically, 1μg of genomic DNA was sheared by Covaris^®^ sonication and fragment size was determined by Lonza Flash Gel Cassette. DNA fragments were end-repaired and A-tailed using the appropriate NEBNext reaction modules according to the manufacturer’s protocols. Illumina multiplex adapter ligation was followed by PCR amplification using modified barcode primers. Agencourt^®^ XP^®^ Beads were used to purify DNA after each enzymatic reaction. PCR product quantification and size distribution were determined using Caliper^®^ GX 1K/12K/High Sensitivity Assay Labchips. Precapture libraries (1μg) were hybridized in solution to custom-designed NimbleGen Solution Probes according to the manufacturer’s protocol, except that hybridization enhancing oligos IHE1, IHE2 and IHE3 replaced the HE1.1, and HE2.1 oligos, and post-capture LM-PCR was performed using 14 cycles. Probes were designed across chromosome 4 (56761923-157261923bp) and chromosome 16 (49433318-50080204bp) (hg19) and were successfully produced across 93% of the target intervals. Capture libraries were quantified using Caliper^®^ Labchips, and capture QC was evaluated using qPCR and built-in control oligo sets. The post-capture libraries were amplified and clusters were generated using the illumina cBot^®^ system according to the manufacturer’s protocols. Sequencing was performed in paired-end mode with the Illumina HiSeq 2000, and all lanes were spiked with a 1% phiX control library.

At Baylor, all sequencing reads that passed the Illumina Chastity filter were formatted into fastq files and aligned to the genome using BWA against human genome build #19 with default parameters, except for a 40bp seed sequence. Two seed mismatches, and a total of 3 mismatches were allowed. Base qualities of aligned reads were recalibrated, alignments were refined using GATK ([1](#_ENREF_1), [2](#_ENREF_2)), and raw variants were extracted using AtlasSNP and were stored in Variant Call Format (VCF). VCF files were annotated as somatic, germline or unknown, and each variant was assessed for relevant characteristics including frequency, base coverage in each genome, base quality, mapping quality of reads harboring variants and read orientation. Potential variant impact on gene function was annotated, e.g., missense, nonsense, splice-site events etc. All variants were also annotated by comparison to dbSNP, RefSeq, and Interpro.

At Mayo, the BAM files produced by the Baylor College of Medicine Human Genome Sequencing Center (BCM-HGSC) were analyzed using TREAT (Targeted RE-sequencing and Annotation Tool), a workflow developed at Mayo for sequence alignment, variant calling, functional prediction, and variant annotation ([2](#_ENREF_2)). The overall workflow is depicted graphically in **Supplementary Figure 1**. Briefly, reads were aligned to the human reference genome using BWA and duplicate reads were removed using Picard’s MarkDuplicates (<http://picard.sourceforge.net/>). The BWA alignment was then improved using the Genome Analysis ToolKit (GATK) local indel realigner and base quality score recalibrator ([1](#_ENREF_1)). SNVs were called using SNVMix ([3](#_ENREF_3)), and indels were called using GATK’s UnifiedGenotyper with default parameter settings. The SNVMix posterior probability threshold (0.8) was chosen based on analysis of a CEU sample sequenced by the 1000 Genomes Project ([4](#_ENREF_4)). All variants located within the target regions were retained. SIFT ([5](#_ENREF_5)) and SeattleSeq (http://gvs.gs.washington.edu/SeattleSeqAnnotation131) were used for functional annotation. The number of supporting reads for each A, C, G, and T base at each variant position, as well as the number of forward and reverse mapping reads were generated by curating the BAM pile-up files using SAMtools ([6](#_ENREF_6)). Potential splice variants were defined as those within 2bp of an exon-intron boundary.

Sequencing data were processed to exclude reads and variants with poor mapping and quality scores. Reads with a mapping quality (Qphred) score of <20 were excluded from analysis, and any variant that did not meet a minimum cut-off probability score (0.8) was also excluded from further analysis. Additionally, we required that there be a minimum of 10 high-quality variant supporting reads for each SNV and a minimum of 3 high-quality variant supporting reads for each indel to reduce the number of false positives. During read alignment, we identified several reads that aligned to off-target regions with high mapping scores. Therefore, we expanded the target region to include the capture region plus all exons.

**Statistical analyses**

The frequencies of variants detected by sequencing were compared between cases and controls in two different ways. The first method, a “weighted analysis”, used the inverse of the design-based sampling fractions as weights. By stratifying on the SNPs observed during the initial GWAS, and then using inverse sampling fractions as weights, there was little loss of statistical efficiency for variants detected by sequencing that were correlated with the GWAS SNPs—almost as if the variants detected by sequencing were measured on the entire sample used in the GWAS. By use of the weighted analysis, we could evaluate the association of newly detected variants with case-control status to approximate what would have been observed if every sample included in our GWAS were sequenced, at least for variants highly correlated with the GWAS SNPs on which we stratified. For uncorrelated variants, the associations are also valid, but would have larger variance due to a smaller effective sample size.

Our second method of analysis was a traditional “unweighted” logistic regression. Because we balanced cases and controls across all of the sampling strata defined by the GWAS SNPs, the unweighted analysis implicitly adjusted out the effects of the GWAS SNPs used to create the strata. That is, the unweighted analysis was a design-based adjustment of the initial GWAS SNPs, so that any associations of new variants detected from sequencing based on case-control status were the effects of those variants, adjusted for the GWAS SNPs.

**DNA sequence variation across *CTSO* and *ZNF423***

The procedure used to choose the 400 DNA samples selected for resequencing, as described in detail in the **Methods**, is illustrated in **Supplementary Table 5,** each row in the table represents the joint genotypes of the rs6835859 GWAS SNP on chromosome 4 and the rs8060157 GWAS SNP on chromosome 16, the top genotyped SNPs observed in terms of *p* values for these two signals ([7](#_ENREF_7)). The total numbers of cases and controls genotyped in each strata during our initial GWAS are listed in the columns labeled “GWAS Samples”. The number of randomly sampled participants selected for resequencing is listed in the columns labeled “Sequenced Samples”. The sampling weights used in the analyses are listed in the final columns, computed as the ratio of the GWAS sample count divided by the resequenced sample count. The clinical characteristics of the 400 participants for whom we resequenced DNA are listed in **Supplementary Table 2** The target region resequenced on chromosome 4 covered 499,868bp with 4,488 SNP positions, and the target region resequenced on chromosome 16 was 550,164bp in length, with 4,553 SNP positions. For both target regions, the depth of coverage was high, with a median depth, over all subjects and all positions, of 291X and 299X for the chromosome 4 and 16 regions, respectively.

The nucleotide variants were filtered based on whether they were monomorphic, missing in more than 10% of participants, or represented genotype calls that were discordant between the Baylor and Mayo TREAT workflows (see **Supplementary Figure 1**). By requiring 100% agreement between the Baylor and the Mayo TREAT workflow genotype calls, we retained the highest quality variants for analyses, even though many of the discordant variants had concordance rates in the 80-99% range. As a result, the final number of analyzed variants was 3,876 for chromosome 4 and 4,079 for chromosome 16. The fraction of very rare variants (MAF ≤0.005) was 50% for chromosome 4 and 60% for chromosome 16. The percentages of less common variants (MAF 0.005–0.01) were 3.4% for chromosome 4 and 4.8% for chromosome 16, and the percentages of variants with MAF >0.01 were 46.6% for chromosome 4 and 35.1% for chromosome 16.

Results obtained by comparing the frequencies of variants between cases and controls on the chromosome 4 target region are shown in graphically **Supplementary Figure 2**. The weighted analysis shown in **Supplementary Figure 2A** emphasizes newly discovered variants that were correlated with the initial GWAS top genotyped SNP, rs6835859, depicted in **Supplementary Figure 2A** by the “G” (result for rs6835859), and the association signals directly beneath it. In contrast, the unweighted analysis in **Supplementary Figure 2B** shows associations for newly discovered variants after adjusting for the effect of the rs6835859 SNP.

Similar association results for the chromosome 16 target region are shown in **Supplementary Figure 3**. The unweighted analyses replicated the original GWAS association signal (“G” in **Supplementary Figure 3A** represents the top GWAS genotyped SNP, rs8060157), with a few additional variants detected by sequencing that were in strong linkage disequilibrium (LD) with rs8060157. The unweighted analyses in **Supplementary Figure 3B** show association signals in intron 5 at the 3’-end of the *ZNF423* gene at approximately 49.6MB. We will return to two of those SNPs, rs746157 and rs12918288, subsequently because of striking findings for these two SNPs during the functional genomic experiments.

**References**

1. McKenna A, Hanna M, Banks E, Sivachenko A, Cibulskis K, Kernytsky A, et al. The Genome Analysis Toolkit: a MapReduce framework for analyzing next-generation DNA sequencing data. Genome Res. 2010;20(9):1297-303.

2. Asmann YW, Middha S, Hossain A, Baheti S, Li Y, Chai H-S, et al. TREAT: a bioinformatics tool for variant annotations and visualizations in targeted and exome sequencing data. Bioinformatics. 2012 January 15, 2012;28(2):277-8.

3. Goya R, Sun MGF, Morin RD, Leung G, Ha G, Wiegand KC, et al. SNVMix: predicting single nucleotide variants from next-generation sequencing of tumors. Bioinformatics. 2010 March 15, 2010;26(6):730-6.

4. A map of human genome variation from population-scale sequencing. Nature. [10.1038/nature09534]. 2010;467(7319):1061-73.

5. Irizarry RA, Bolstad BM, Collin F, Cope LM, Hobbs B, Speed TP. Summaries of Affymetrix GeneChip probe level data. Nucleic Acids Res. 2003 February 15, 2003;31(4):e15.

6. Li H, Handsaker B, Wysoker A, Fennell T, Ruan J, Homer N, et al. The Sequence Alignment/Map format and SAMtools. Bioinformatics. 2009 August 15, 2009;25(16):2078-9.

7. Ingle JN, Liu M, Wickerham DL, Schaid DJ, Wang L, Mushiroda T, et al. Selective estrogen receptor modulators and pharmacogenomic variation in ZNF423 regulation of BRCA1 expression: individualized breast cancer prevention. Cancer discovery. 2013 Jul;3(7):812-25.
