# Supplementary material for: Breast cancer chemoprevention pharmacogenomics: Deep sequencing and functional genomics of the ZNF423 and CTSO genes
Source: NPJ Breast Cancer. 2017 Aug 21;3:30. doi: 10.1038/s41523-017-0036-4 (PMC5566425; doi:10.1038/s41523-017-0036-4)
Supplement: Supplementary file 2 — Supplementary Tables [file 41523_2017_36_MOESM2_ESM.docx]

| **Supplementary Table 1**. *ZNF423* and *CTSO* nsSNPs and the primers for site-directed mutagenesis. | | | | |
| --- | --- | --- | --- | --- |
| **Gene** | **Amino Acid Substitution** | **SNPs** | **Forward Primer** | **Reverse Primer** |
| ***ZNF423*** | S382P | 1144 T>C | CTGTGGAGCGTGGCcCCACCCCGGACTC | GAGTCCGGGGTGGgGCCACGCTCCACAG |
|  | R417W | 1249 C>T | GCCCCTATTGTTCCAAGtGGGACTTTAACAGCCTG | CAGGCTGTTAAAGTCCCaCTTGGAACAATAGGGGC |
|  | V460I | 1378 G>A | CAACCTCAACGAGCACaTTCGCAAGCTGCACAAG | CTTGTGCAGCTTGCGAAtGTGCTCGTTGAGGTTG |
|  | R617Q | 1850 G>A | GTCTTCCCCGAAGCaGCAGCGGCTCTCAG | CTGAGAGCCGCTGCtGCTTCGGGGAAGAC |
|  | N629S | 1886 A>G | CCAACTCCATCTCCAgTGGGGAGTATCCTTG | CAAGGATACTCCCCAcTGGAGATGGAGTTGG |
|  | T723I | 2168 C>T | GTGTTGTACCACTGCAtCCTGTGTCAGGAGGTC | GACCTCCTGACACAGGaTGCAGTGGTACAACAC |
|  | A836V | 2508 C>T | GTGTGTTTGATGCTGtGACCGAGAACGGCAC | GTGCCGTTCTCGGTCaCAGCATCAAACACAC |
|  | G887S | 2659 G>A | GTCGGAGCCCATGTACaGCTGTGACATCTGCG | CGCAGATGTCACAGCtGTACATGGGCTCCGAC |
|  | A1047V | 3140 C>T | CATGCAGAAGCTGGtGGGCAGCTCAGCGG | CCGCTGAGCTGCCCaCCAGCTTCTGCATG |
|  | A1113T | 3337 G>A | CCCGCCCGAGCCCaCCGACCGGCCCTG | CAGGGCCGGTCGGtGGGCTCGGGCGGG |
| ***CTSO*** | S208F | 623 C>T | ATGGTCTGTGCCATTACTTTTtTGGTTCACATTCTGGATTTTC | GAAAATCCAGAATGTGAACCAaAAAAGTAATGGCACAGACCAT |

**Supplementary Table 2**. Clinical characteristics of the 400 participants whose DNA was used in this deep sequencing study.

| **Supplementary Table 2** | | |
| --- | --- | --- |
| **Summary of NSABP Clinical Data** | | |
|  | Controls (N=201) | Cases (N=199) |
| **NSABP Trial** |  |  |
| P-1 | 30 (14.9%) | 29 (14.6%) |
| P-2 | 171 (85.1%) | 170 (85.4%) |
|  |  |  |
| **Type of Breast Event** |  |  |
| Missing | --- | 0 |
| Invasive Breast Cancer | --- | 148 (74.4%) |
| DCIS | --- | 51 (25.6%) |
|  |  |  |
| **Estrogen Receptor Status**  **(Invasive Breast Cancer Only)** |  |  |
|  |  |  |
| Negative | 0 (0.0%) | 46 (31.1%) |
| Positive | 0 (0.0%) | 95 (64.2%) |
| Unknown | 0 (0.0%) | 7 (4.7%) |
|  |  |  |
| **Treatment** |  |  |
| Tamoxifen | 111 (55.2%) | 117 (58.8%) |
| Raloxifene | 90 (44.8%) | 82 (41.2%) |
|  |  |  |
| **Age (yrs) at entry** |  |  |
| Mean (SD) | 60.2 (7.1) | 59.5 (6.9) |
| Median | 59.0 | 59.0 |
| Q1, Q3 | 55.0, 66.0 | 55.0, 64.0 |
| Range | (47.0-77.0) | (42.0-77.0) |
|  |  |  |
| **5-year predicted breast cancer risk** |  |  |
| Mean (SD) | 4.8 (2.4) | 5.0 (3.0) |
| Median | 4.0 | 4.6 |
| Q1, Q3 | 3.0, 6.3 | 2.9, 6.3 |
| Range | (1.5-14.8) | (1.2-22.4) |
|  |  |  |
| **History of LCIS at entry** |  |  |
| NO | 167 (83.1%) | 164 (82.4%) |
| YES | 34 (16.9%) | 35 (17.6%) |
|  |  |  |
| **History of Atypical Hyperplasia at entry** |  |  |
| NO | 154 (76.6%) | 154 (77.4%) |
| YES | 47 (23.4%) | 45 (22.6%) |
|  |  |  |
| **History of Hysterectomy at entry** |  |  |
| NO | 100 (49.8%) | 111 (55.8%) |
| YES | 101 (50.2%) | 88 (44.2%) |
|  |  |  |
|  |  |  |
|  |  |  |
| **Number of First-degree Relatives with Breast Cancer** |  |  |
| 0 | 59 (29.4%) | 68 (34.2%) |
| 1 | 99 (49.3%) | 84 (42.2%) |
| 2+ | 43 (21.4%) | 47 (23.6%) |
|  |  |  |
| **BMI** |  |  |
| Mean (SD) | 28.2 (5.8) | 28.5 (6.2) |
| Median | 26.4 | 27.5 |
| Q1  Q3 | 24.2  31.9 | 24.2  31.9 |
| Range | (18.7-56.1) | (17.6-47.3) |
|  |  |  |
| **Smoker at time of entry** |  |  |
| Missing | 1 | 1 |
| NO | 178 (89.0%) | 183 (92.4%) |
| YES | 22 (11.0%) | 15 (7.6%) |
|  |  |  |
| **Hot Flash Score** |  |  |
| Missing | 1 | 5 |
| Not at all | 25 (12.5%) | 36 (18.6%) |
| Slightly | 37 (18.5%) | 27 (13.9%) |
| Moderately | 33 (16.5%) | 41 (21.1%) |
| Quite a bit | 64 (32.0%) | 51 (26.3%) |
| Extremely | 41 (20.5%) | 39 (20.1%) |

**Supplementary Table 3**. SNPs across the *ZNF423* gene on chromosome 16 that were predicted to create or disrupt ERE motifs. p values are for associations with the occurrence of breast cancer during SERM therapy.

| SNP rs ID | SNP Position on Chr.16 | Motif Sequence | Motif Start Position | Motif End Position | Strand | MAF (400) | MAF (all samples) | *p* Value |
| --- | --- | --- | --- | --- | --- | --- | --- | --- |
| rs72780322 | 49690876 | CATGGATTCCCTGACCT | 49690861 | 49690878 | + | 0.01250 | 0.0104025 | 2.39E-02 |
| rs12926400 | 49541976 | GGGGGGTTAGTTGACCT | 49541974 | 49541991 | + | 0.37875 | 0.3409303 | 1.64E-01 |
| rs74606373 | 49706980 | GGTGGTGAGCCTCACCT | 49706964 | 49706981 | - | 0.00250 | 0.0013953 | 1.76E-01 |
| rs9936011 | 49547911 | CAGTGGCCGTCTGTCCT | 49547901 | 49547918 | + | 0.38125 | 0.342876 | 1.76E-01 |
| ------ | 49952373 | CATAGGCCACAGGACCT | 49952371 | 49952388 | - | 0.00875 | 0.0071528 | 2.34E-01 |
| rs8063279 | 49544240 | CTTTGTCAATGTGACCT | 49544223 | 49544240 | - | 0.02250 | 0.0297872 | 2.46E-01 |
| rs8051105 | 49547913 | CAGTGGCCGTCTGTCCT | 49547901 | 49547918 | + | 0.02250 | 0.0297872 | 2.46E-01 |
| ------ | 49759999 | CCGGGGCTGGAGCACTT | 49759984 | 49760001 | - | 0.00125 | 0.0004813 | 3.08E-01 |
| ------ | 49830977 | CAAGGGGCACGTGACTG | 49830967 | 49830984 | + | 0.00125 | 0.0004813 | 3.08E-01 |
| ------ | 49901192 | CTGGGTCTAAAGGACCT | 49901187 | 49901204 | + | 0.00125 | 0.0004813 | 3.08E-01 |
| ------ | 49965618 | CATGAGCAGGGGGACTT | 49965608 | 49965625 | - | 0.00125 | 0.0004813 | 3.08E-01 |
| ------ | 49608508 | GTATGGCAAGCTGTCCC | 49608501 | 49608518 | - | 0.00125 | 0.0006876 | 3.08E-01 |
| ------ | 49830968 | CAAGGGGCACGTGACTG | 49830967 | 49830984 | + | 0.00125 | 0.0010201 | 3.10E-01 |
| ------ | 49626686 | CCAGAACCGGCGGACCT | 49626672 | 49626689 | + | 0.00125 | 0.0015726 | 3.11E-01 |
| ------ | 49809279 | CAAAGGCATCCTGACCC | 49809273 | 49809290 | + | 0.00125 | 0.0015726 | 3.11E-01 |
| ------ | 49915178 | GGAGGGGACCATGACCC | 49915162 | 49915179 | - | 0.00125 | 0.0015726 | 3.11E-01 |
| ------ | 49966185 | GAAGGCCACCATGAATT | 49966179 | 49966196 | - | 0.00125 | 0.0016774 | 3.12E-01 |
| ------ | 49706970 | GGTGGTGAGCCTCACCT | 49706964 | 49706981 | - | 0.00125 | 0.0018411 | 3.12E-01 |
| ------ | 49776039 | AAATGTCAGCCTGTCCT | 49776027 | 49776044 | - | 0.00125 | 0.0018411 | 3.12E-01 |
| ------ | 49776039 | CCAGGACAGGCTGACAT | 49776025 | 49776042 | + | 0.00125 | 0.0018411 | 3.12E-01 |
| ------ | 49544227 | CTTTGTCAATGTGACCT | 49544223 | 49544240 | - | 0.00125 | 0.002435 | 3.14E-01 |
| ------ | 50027018 | AACGGGCATGTTGACCC | 50027013 | 50027030 | - | 0.00125 | 0.002435 | 3.14E-01 |
| rs4785369 | 49969963 | CAGGAGCTGGGGGACTT | 49969957 | 49969974 | - | 0.42875 | 0.4183081 | 3.17E-01 |
| rs80352883 | 49621295 | CCAGGTCTGTGGCACCT | 49621282 | 49621299 | - | 0.00750 | 0.0066864 | 5.04E-01 |
| rs78557935 | 49692071 | CCAGGGCAGCAGGACTT | 49692070 | 49692087 | - | 0.04375 | 0.0461836 | 5.41E-01 |
| ------ | 49592987 | GAAGGGGCAGCTGACTC | 49592974 | 49592991 | - | 0.01625 | 0.017712 | 9.94E-01 |

**Supplementary Table 4**. SNPs across or near the *CTSO* gene on chromosome 4 that were predicted to create or disrupt ERE motifs. *p* values are for associations with the occurrence of breast cancer during SERM therapy.

| SNP rs ID | SNP Position on Chr.4 | Motif Sequence | Motif Start Position | Motif End Position | Strand | MAF (400) | MAF (all samples) | *p* Value |
| --- | --- | --- | --- | --- | --- | --- | --- | --- |
| rs62329278 | 157031765 | CTGGGTCACTATCTCCC | 157031749 | 157031766 | - | 0.02625 | 0.040123 | 7.48E-02 |
| rs75198806 | 157084293 | GAATAGCACTATGACCT | 157084285 | 157084302 | + | 0.06125 | 0.066367 | 7.59E-02 |
| rs55734984 | 157149209 | CGTGAGCCACCTCACCT | 157149201 | 157149218 | - | 0.07375 | 0.075331 | 1.93E-01 |
| rs6536105 | 156765293 | CTGAGTGAAGCTGACCT | 156765282 | 156765299 | + | 0.00250 | 0.00397 | 2.21E-01 |
| rs6536105 | 156765293 | CCAGGTCAGCTTCACTC | 156765284 | 156765301 | - | 0.00250 | 0.00397 | 2.21E-01 |
| ------ | 157032001 | GAGGGCCAACTTGTCTC | 157031985 | 157032002 | + | 0.00125 | 0.000457 | 3.07E-01 |
| rs117256944 | 157052986 | GCAGGGCTGCCTGACCT | 157052974 | 157052991 | + | 0.00125 | 0.000481 | 3.08E-01 |
| ------ | 157092153 | GATGGTCTCGATCTCCT | 157092144 | 157092161 | - | 0.00125 | 0.000645 | 3.08E-01 |
| rs2011902 | 156789601 | TAGGATCTTGGTGTCCT | 156789597 | 156789614 | - | 0.00125 | 0.000688 | 3.08E-01 |
| ------ | 157101817 | GCTGGGCCAAATGACTT | 157101816 | 157101833 | + | 0.00125 | 0.000926 | 3.09E-01 |
| ------ | 157037628 | GAATGTCTAGATGACAC | 157037621 | 157037638 | - | 0.00125 | 0.001841 | 3.12E-01 |
| ------ | 156966086 | GTGGGTCTGCCTGTCCC | 156966075 | 156966092 | + | 0.00125 | 0.002435 | 3.14E-01 |
| ------ | 156895311 | TGATATCAGCCTCACCT | 156895298 | 156895315 | + | 0.01000 | 0.014488 | 3.78E-01 |
| rs75637798 | 156918520 | TTAGGGTAGAATGACTC | 156918505 | 156918522 | + | 0.02375 | 0.038665 | 5.57E-01 |
| rs55696238 | 156956564 | CCTGTGCAGGATGACTT | 156956555 | 156956572 | - | 0.29875 | 0.252112 | 6.77E-01 |
| rs7661151 | 157065076 | AATGTTCATCCTGACCT | 157065073 | 157065090 | + | 0.39500 | 0.432938 | 8.16E-01 |
| rs114863852 | 156804999 | GAGAGTCCACTTGACAT | 156804996 | 156805013 | + | 0.04875 | 0.058451 | 9.07E-01 |
| rs114863852 | 156804999 | GAATGTCAAGTGGACTC | 156804998 | 156805015 | - | 0.04875 | 0.058451 | 9.07E-01 |

**Supplementary Table 5**. Sampling design and weights of samples.

| Chromosome Genotypes | |  | GWAS Samples | |  | Sequenced Samples | |  | Sampling Weights | |
| --- | --- | --- | --- | --- | --- | --- | --- | --- | --- | --- |
| 4* | 16* |  | Controls | Cases |  | Controls | Cases |  | Controls | Cases |
| 0 | 0 |  | 117 | 79 |  | 22 | 22 |  | 5.32 | 3.59 |
| 0 | 1 |  | 244 | 78 |  | 22 | 22 |  | 11.09 | 3.55 |
| 0 | 2 |  | 112 | 19 |  | 22 | 19 |  | 5.09 | 1.00 |
| 1 | 0 |  | 158 | 105 |  | 22 | 22 |  | 7.18 | 4.77 |
| 1 | 1 |  | 277 | 150 |  | 22 | 22 |  | 12.59 | 6.82 |
| 1 | 2 |  | 113 | 48 |  | 22 | 23 |  | 5.14 | 2.09 |
| 2 | 0 |  | 41 | 42 |  | 23 | 23 |  | 1.78 | 1.83 |
| 2 | 1 |  | 74 | 45 |  | 23 | 23 |  | 3.22 | 1.96 |
| 2 | 2 |  | 31 | 25 |  | 23 | 23 |  | 1.35 | 1.09 |

Each row in the table represents the joint genotypes of the rs6835859 GWAS SNP on chromosome 4 and the rs8060157 GWAS SNP on chromosome 16, the top genotyped SNPs observed in terms of *p* values for these two signals. The rs8060157 SNP was in tight LD with rs9940645 (r^2^=0.99). The total numbers of cases and controls genotyped in each strata during our initial GWAS are listed in the columns labeled “GWAS Samples”. The number of randomly sampled participants selected for resequencing is listed in the columns labeled “Sequenced Samples”. The sampling weights used in the analyses are listed in the final columns, computed as the ratio of the GWAS sample count divided by the resequenced sample count. Please see the **Supplementary Methods** for additional details.

**Supplementary Table 6**. Primers used for ChIP assays to amplify fragments containing *ZNF423* and *CTSO* SNPs.

| Gene | rs ID | Forward Primer | Reverse Primer |
| --- | --- | --- | --- |
| *ZNF423* | rs7187662 | TCAGTCCTGCGAGATGACC | GATATTGGGCCTTCCAGGAT |
| *ZNF423* | rs72780324 | AGGGCCTTACAGGTGGAGAT | AGGGAGTGTTTTTGGGGAGT |
| *ZNF423* | rs746157 | TTCCCTCAACCTTGGACATC | TCCTCTTGGGGCAGAGATAA |
| *ZNF423* | rs12925456 | GAATCAGACCAGATGATTTGAGG | GTGGCTCATGCCTGTAATTC |
| *ZNF423* | rs11642983 | AGAGATGGGGTTTCACCATG | TGCCCCACTTTCCTTGACTA |
| *ZNF423* | rs72780328 | ATGTGTACACGGTGCGTCAT | GCCCTGACTGGTTTTGTGTT |
| *ZNF423* | rs71382759 | CAGCCAGTGAGAAGGGAGAC | TGGGAAATGAAAGGATCAGC |
| *ZNF423* | rs12918288 | TTCCCTCAACCTTGGACATC | GAGAAGGCTTTGCTTTTCTG |
| *ZNF423* | rs57148286 | TCTGGGCCTACAGAAGATGG | ACAGCATGAGCTCCTTGTCC |
| *CTSO* | rs1490555 | TTGCAGGTGTTTAGCAGCTG | TGTGAGGCTTAATGACGTGC |
| *CTSO* | rs1490556 | TTGCAGGTGTTTAGCAGCTG | TAACCAATCCAGTGTCGCAA |
| *CTSO* | rs1873358 | TTGCAGGTGTTTAGCAGCTG | TCCAGACTCTCTTGGGCTTG |
| *CTSO* | rs2879978 | TTGCAGGTGTTTAGCAGCTG | CCCACACACCCTTTCAAATC |
| *CTSO* | rs10010729 | GTCCAGGTAGCAGTGTGCAA | GGCACAGATGCTGTTTTCTC |
| *CTSO* | rs1490554 | TTGCAGGTGTTTAGCAGCTG | TTAGGAAAGTAAACCCAGTGCTG |
| *CTSO* | rs2101586 | TTAGGCAAACTTGGGCTTTG | GGTTTCATGTACAATGCAGGTC |
| *CTSO* | rs6536168 | CAGCCTGGGTTTACATGACC | GGCACTACCAAGTCAAAACCA |
| *CTSO* | rs4691210 | AATTCACAAGCAACGGATGG | GTTGGGGTATGAGCAAATTG |
| *CTSO* | rs4691214 | GACCCAGAAGTCCATTCTTAAAC | TTCTCCAAGTCCCCTCACTC |
| *CTSO* | rs4691216 | GGGCACAAATAAGGGAAGAA | CATGGTCTCGCTGACTTCAA |
| *CTSO* | rs4234895 | TTGCTGCTGCTCACTCTTTG | GAGAAGAAGAATCTTGATTCAGGA |
