# Supplementary material for: Breast cancer chemoprevention pharmacogenomics: Deep sequencing and functional genomics of the ZNF423 and CTSO genes
Source: NPJ Breast Cancer. 2017 Aug 21;3:30. doi: 10.1038/s41523-017-0036-4 (PMC5566425; doi:10.1038/s41523-017-0036-4)
Supplement: Supplementary file 3 — Supplementary Figures [file 41523_2017_36_MOESM3_ESM.pdf]

Supplementary Figure 1

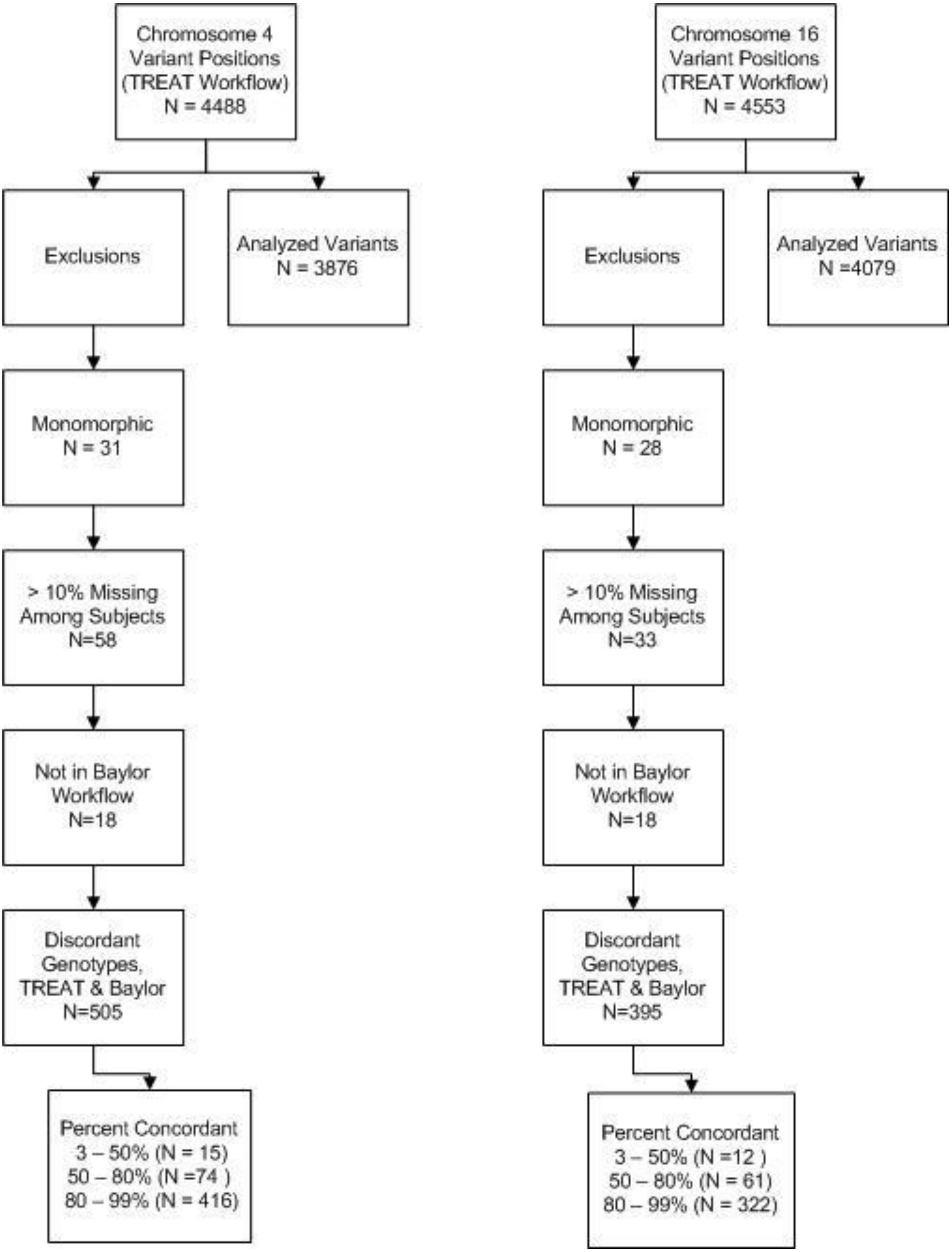

Supplementary Figure 1. The figure shows schematically the Mayo Clinic TREAT workflow for the chromosome 4 and 16 Next Generation DNA sequencing data and a comparison with the results of the BCM-HGSC workflow results.

Supplementary Figure 2

(a) Chromosome 4 weighted association analysis. "G" represents GWAS results for the rs6835859 .

(b) Chromosome 4 unweighted association analysis, adjusted for the rs6835859.

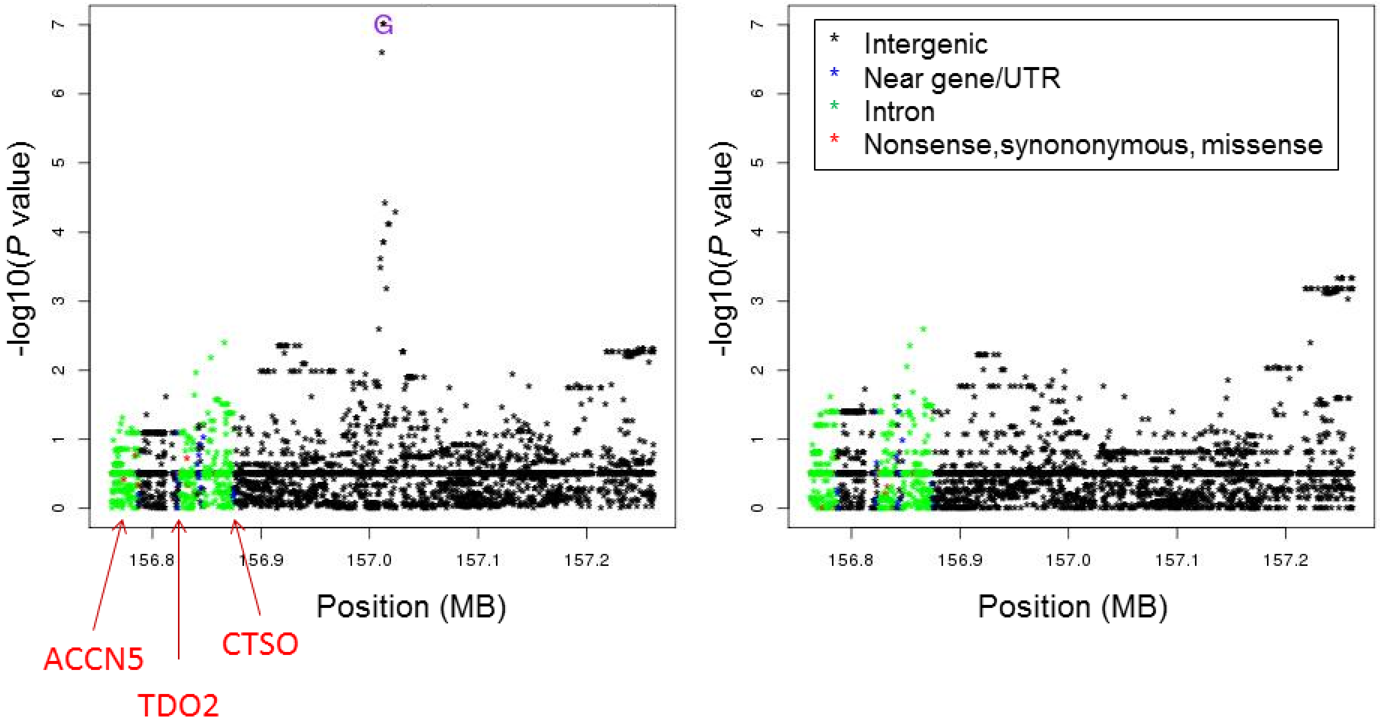

Supplementary Figure 2. The figure shows the results of weighted (A) and unweighted (B) association analyses across the area of chromosome 4 surrounding the rs6835859 SNP 5' of the *CTSO* gene. Locations of the *CTSO*, *TDO2* and *ACCN5* genes are indicated. See text for details.

Supplementary Figure 3

(a) Chromosome 16 weighted association analysis. “G” represents GWAS results for the rs8060157 SNP.

(b) Chromosome 16 unweighted association analysis, adjusted for the GWAS rs8060157 SNP.

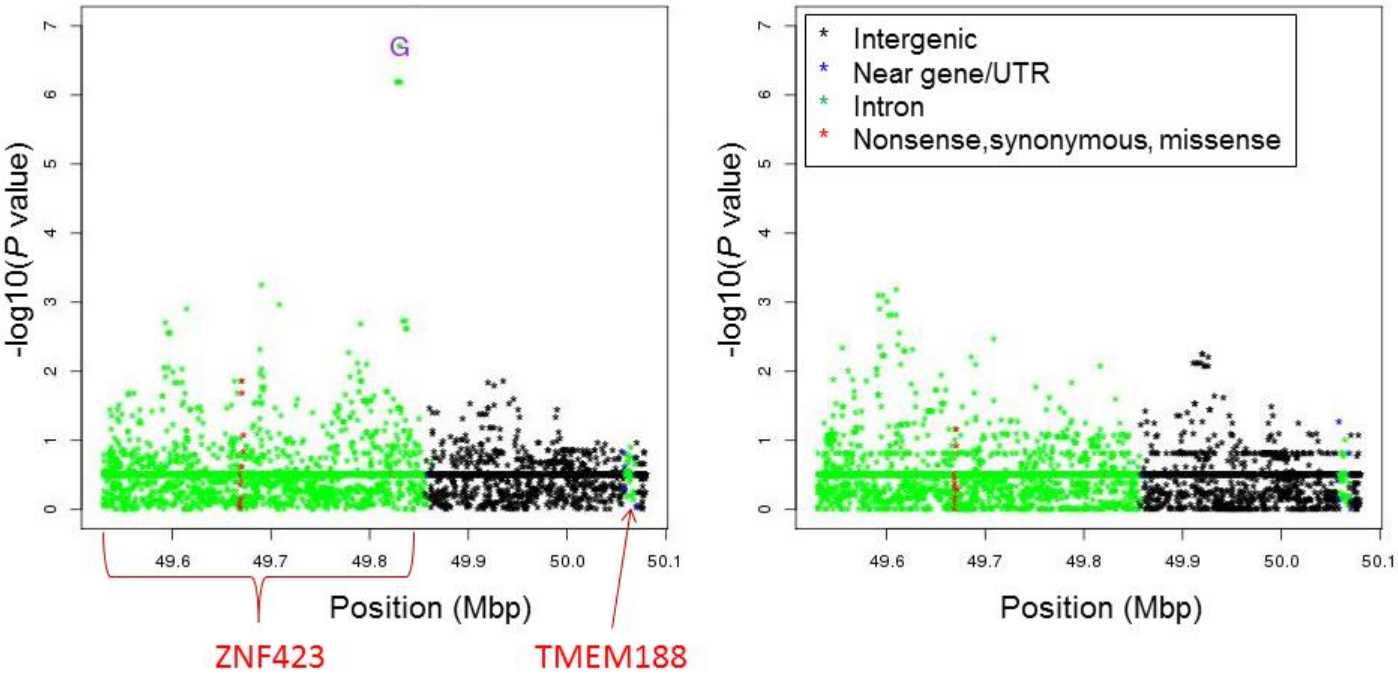

Supplementary Figure 3. The figure shows the results of weighted (A) and unweighted (B) association analyses across the area of chromosome 16 surrounding the rs8060157 SNP and across *ZNF423*. Locations of the *ZNF423* and *TMEM188* genes are indicated. See text for details.

Supplementary Figure 4

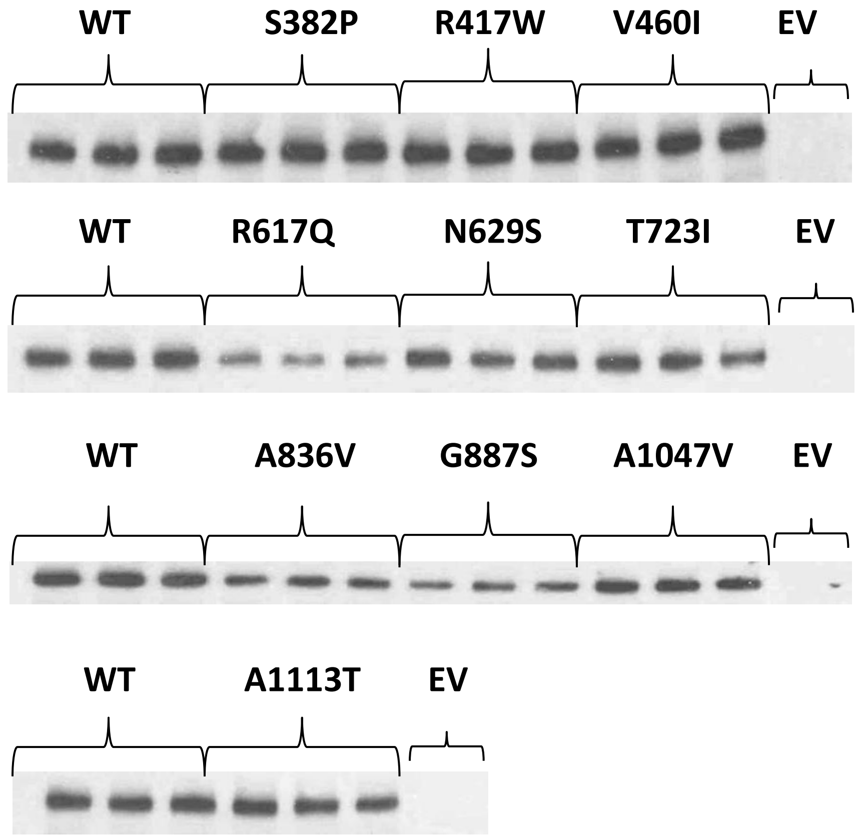

Supplementary Figure 4. Western blot analysis of ten ZNF423 variant allozymes expressed in COS-1 cells as compared with WT allozyme. A typical quantitative Western blot analysis of wild type (WT) and variant allozymes is shown. WT samples were run on each gel to serve as a standard. “EV” is “empty vector”. All samples were run in triplicate.

Supplementary Figure 5

(a)

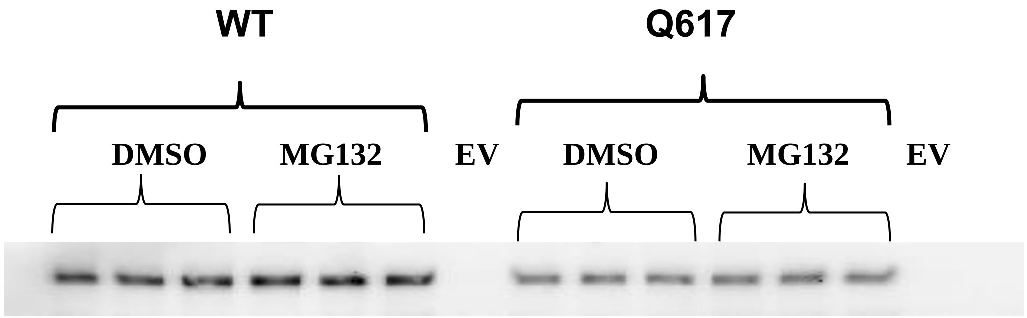

(b)

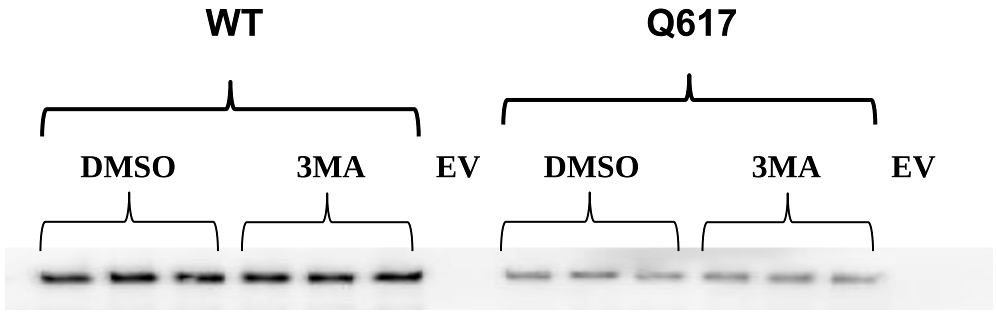

Supplementary Figure 5. Western blot analysis of ZNF423 WT and Q617 variant allozyme protein after expression in COS-1 cells showing treatment with the proteasome inhibitor MG132 and the autophagy inhibitor 3MA. (A) Comparison of the expression of WT ZNF423 and the Q617 variant allozyme after treatment with DMSO or MG132 dissolved in DMSO. After MG132 treatment, the Q617 variant protein level was not significantly changed compared to the DMSO treated samples. (B) Comparison of the expression of WT ZNF423 and the Q617 variant protein after treatment with DMSO or 3MA, respectively. 3MA treatment did not result in a significant change in Q617 variant protein expression.

Supplementary Figure 6

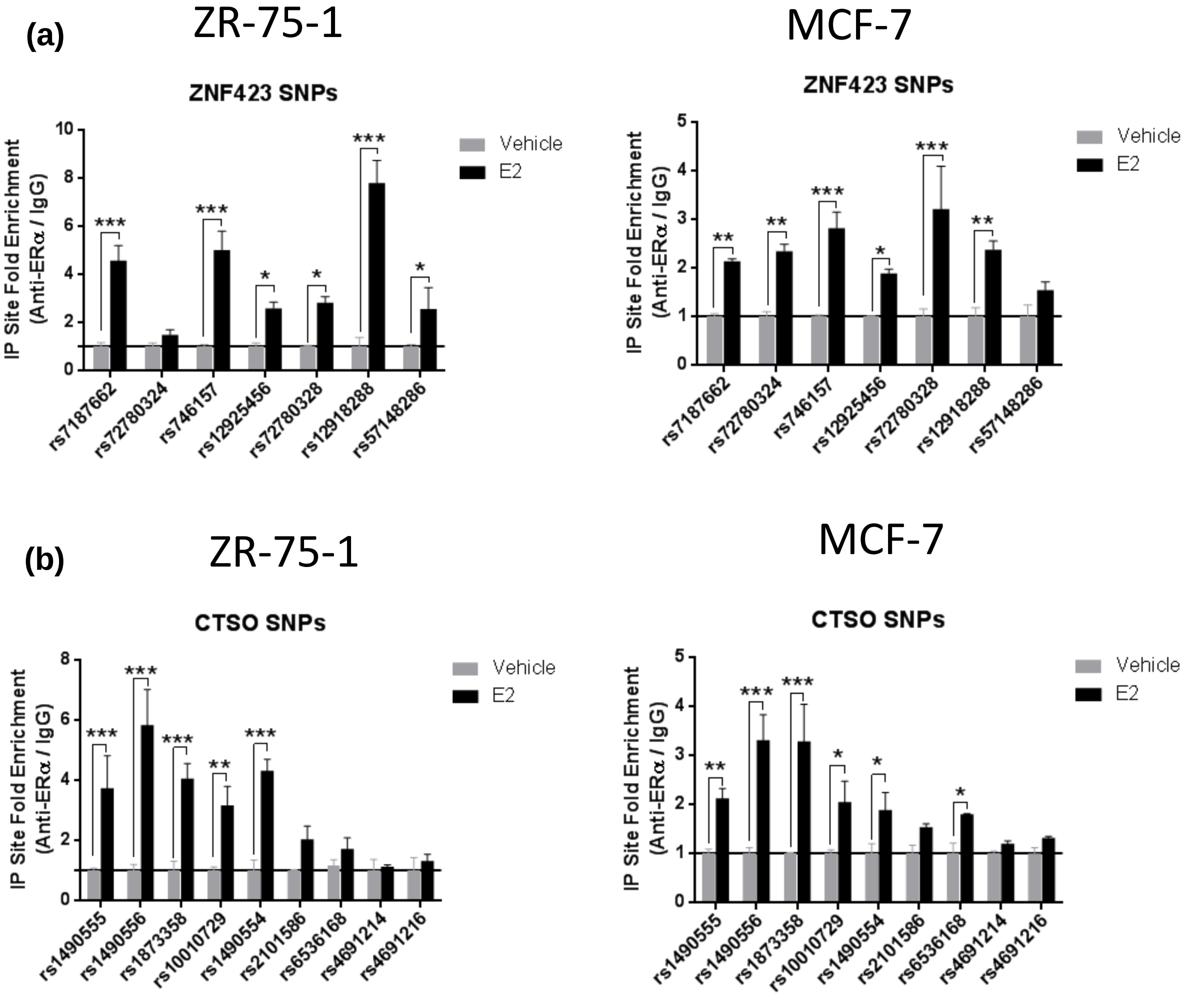

Supplementary Figure 6. ChIP assays showing fold changes in ERα binding to DNA sequences containing *ZNF423* SNPs (panels **A**) or *CTSO* SNPs (panel **B**) in ZR-75-1 and MCF7 breast cancer cell lines. ChIP assays were performed in ZR-75 cells after exposure to vehicle or E2. The values shown represent mean ± SEM for three determinations.**P<0*.05, ***P*<0.01, ***p<0.0001 v.s vehicle. (note: panel (A): both ZR-75-1 and MCF-7 were homozygous wild-type SNP genotypes for the ZNF423 SNPs. The pattern of ERa binding was comparable to the data obtain using LCLs that were homozygous wild-type genotype for those SNPs, as shown in Figure 2A. Panel (B): rs10010729 and rs1490554 were homozygous variant SNP genotypes for the *CTSO* SNPs in ZR-75-1 and the reminder of the SNPs were homozygous wild-type genotypes. However, in MCF-7 cells, rs10010729 , rs1490554, rs2101586, and rs6536168 in *CTSO* were heterozygous, while the reminder were homozygous wild-type genotypes.)
